# Exposure to low-level ambient air pollution and the relationship with lung and bladder cancer in older men, in Perth, Western Australia

**DOI:** 10.1038/s41416-023-02411-x

**Published:** 2023-09-08

**Authors:** Elizabeth H. Lim, Peter Franklin, Michelle L. Trevenen, Mark Nieuwenhuijsen, Bu B. Yeap, Osvaldo P. Almeida, Graeme J. Hankey, Jonathan Golledge, Christopher Etherton-Beer, Leon Flicker, Suzanne Robinson, Jane Heyworth

**Affiliations:** 1 School of Population and Global Health, The University of Western Australia, Crawley, WA Australia; 2 Western Australian Centre for Health and Ageing, The University of Western Australia, Crawley, WA Australia; 3 Barcelona Institute for Global Health – Campus MAR, Barcelona Biomedical Research Park, Barcelona, Spain; 4 Medical School, The University of Western Australia, Crawley, WA Australia; 5 Department of Endocrinology and Diabetes, Fiona Stanley Hospital, Perth, WA Australia; 6 Perron Institute for Neurological and Translational Science, Perth, WA Australia; 7 Queensland Research Centre for Peripheral Vascular Disease, James Cook University and Townsville University Hospital, Townsville, QLD Australia; 8 Curtin School of Population Health, Curtin University, Perth, WA Australia; 9 Deakin Health Economics, Institute for Health Transformation, Deakin University, Burwood, VIC Australia

**Keywords:** Epidemiology, Cancer epidemiology, Lung cancer, Bladder cancer

## Abstract

**Background:**

Air pollution is a cause of lung cancer and is associated with bladder cancer. However, the relationship between air pollution and these cancers in regions of low pollution is unclear. We investigated associations between fine particulate matter (PM_2.5_), nitrogen dioxide, and black carbon (BC), and both these cancers in a low-pollution city.

**Methods:**

A cohort of 11,679 men ≥65 years old in Perth (Western Australia) were followed from 1996–1999 until 2018. Pollutant concentrations, as a time-varying variable, were estimated at participants’ residential addresses using land use regression models. Incident lung and bladder cancer were identified through the Western Australian Cancer Registry. Risks were estimated using Cox proportional-hazard models (age as the timescale), adjusting for smoking, socioeconomic status, and co-pollutants.

**Results:**

Lung cancer was associated with PM_2.5_ and BC in the adjusted single-pollutant models. A weak positive association was observed between ambient air pollution and squamous cell lung carcinoma but not lung adenocarcinoma. Positive associations were observed with bladder cancer, although these were not statistically significant. Associations were attenuated in two-pollutant models.

**Conclusion:**

Low-level ambient air pollution is associated with lung, and possibly bladder, cancer among older men, suggesting there is no known safe level for air pollution as a carcinogen.

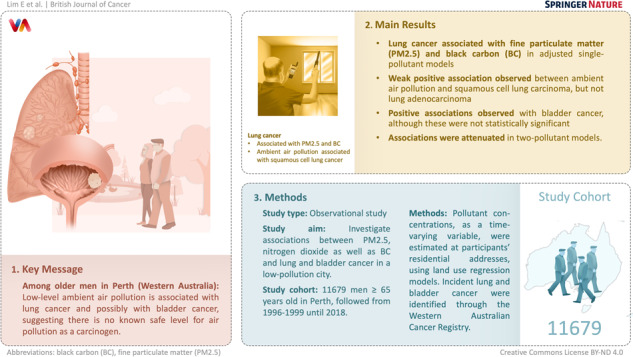

## Background

Lung and bladder cancers are significant causes of mortality and morbidity in Australia and worldwide [1–3]. Ambient air pollution is a known lung carcinogen and possible bladder carcinogen, but the risk in regions with low pollution is unclear.

### Ambient air pollution and lung cancer

Lung cancer is the fifth highest cancer diagnosis and a leading cause of cancer-attributable mortality in Australia [2]. Whilst tobacco smoking remains the strongest known risk factor for lung cancer, international studies have observed that the proportion of non-smoking-related lung cancers is increasing [4–7]. Ambient air pollution may be contributing to this increase [8, 9]. In 2013, ambient air pollution and particulate matter, independently, were classified as Group 1 carcinogens for lung cancer by the International Agency for Research on Cancer (IARC) [10]. The relationship has been confirmed in further meta-analyses [11–13]. Some studies have observed increased lung cancer risks in regions where pollution concentrations were below the region-specific air quality guidelines, and suggested that even low concentrations of air pollution increase the risk of lung cancer [14–19]. However, other studies in low-pollution regions have not shown an increased risk [20–22].

The association between other pollutants and lung cancer is less clear. Significant associations between nitrogen dioxide (NO_2_) and lung cancer have been identified in regions in Europe [23–25], and North America [16, 19, 26], but the data are not consistent [14, 27, 28]. No associations were observed for studies conducted in China [29], Korea [30, 31], and Iran [32]. Studies between black carbon (BC) and lung cancer have also produced inconsistent results [14, 23, 25, 33].

Some studies have investigated associations between pollutants and histological lung cancer subtypes, such as adenocarcinoma and squamous cell carcinoma (SCC), but these are conflicting [14, 18, 23]. Significant associations were observed between (fine particulate matter (PM_2.5_) and adenocarcinoma, but not other subtypes [14, 18]; however, further studies are required [12, 34]. No association was observed between NO_2_ and SCC or adenocarcinoma [14, 30], while studies investigating other air pollutants and different histological subtypes of lung cancer have also found that there were no significant associations [14, 23].

### Ambient air pollution and bladder cancer

Bladder cancer accounts for approximately 2.0% of all new cancer diagnoses in Australia [3]. In 2013, the IARC concluded that there was a possible association between air pollution and bladder cancer, but epidemiological evidence was limited [10]. Associations between PM_2.5_ and bladder cancer have been observed [20, 27, 35–37], but most of these were not statistically significant. No association between PM_2.5_ and bladder cancer was reported in a pooled cohort study in Europe [38]. Studies investigating associations between NO_2_ or BC and bladder cancer are scarce. No associations between NO_2_ and bladder cancer were observed in studies conducted in Europe [36, 37] and the United State of America (USA) [27]. Similarly, no association between BC and bladder cancer was observed in recent studies in Europe [36, 38].

The evidence that a broad range of adverse health effects occurring in regions of low concentrations of pollution is increasing, which resulted in the World Health Organization (WHO) lowering their targets for several air pollutants in the 2021 publication of The WHO Air Quality Guidelines [9]. However, the cancer risks of low-pollution exposure remain unclear. Ambient air pollution in Australian metropolitan cities is generally low [39] and, therefore, conducting studies in Australia provides an opportunity to study cancer incidence in a low-pollution region.

Our primary aims were to investigate whether exposures to low concentrations of air pollutants, specifically PM_2.5_, NO_2_, and BC, were associated with an increased risk of lung and bladder cancer incidence in older men (≥65 years). Our secondary aim was to investigate if exposures to these pollutants were associated with histological subtypes of lung cancer, specifically adenocarcinoma and SCC.

## Methods

### Study design and participants

Participants were members of an ongoing cohort study, the Health In Men Study (HIMS) [40]. The HIMS recruited 12,203 men aged between 65 and 84 years old, living in metropolitan Perth, the capital city of Western Australia (WA), from April 1996 to January 1999 [40]. The men were recruited from the Australian electoral roll; voting is compulsory in Australia and all Australian citizens ≥18 years are required to register on the electoral roll. The HIMS have been followed since 1996, both actively (questionnaires and health assessments) and passively (via the WA Data Linkage Systems (WADLS)) [40]. Using the WADLS, the cohort is linked to several health data collections including the WA Cancer Registry and Mortality Register. Written consent was obtained by all participants, and ethics approval was granted by the Department of Health WA Human Research Ethics Committee, and the University of WA Human Research Ethics Committee.

### Assessment of exposure

We estimated concentrations of PM_2.5_, NO_2_ and BC at each participants’ household address at recruitment using validated Land Use Regression (LUR) models, which are described in detail elsewhere [41, 42]. The development of LUR models followed the protocols of the ESCAPE study [43]. Extensive seasonal air monitoring of PM_2.5_, NO_2_ and BC across the Perth Metropolitan area was conducted in 2012, through a purpose-designed air monitoring campaign. The LUR models were developed to predict the measured concentrations of PM_2.5_, NO_2_ and BC from environmental predictor variables, including road traffic type and volume, open green spaces, land use, buildings, population density, as well as water bodies [42]. The LUR models were in turn used to predict the air pollution concentrations at each participants’ household recruitment address.

Concentrations of PM_2.5_ and NO_2_ from 1995 to 2011 were estimated through back-extrapolation, based on the 2012 LUR model and the fixed air monitoring sites in Perth Metropolitan region, maintained by the WA Department of Water and Environmental Regulation. Exposure estimates from 2013 to 2019 were calculated using a similar method but with forward-extrapolation from the LUR model. Extrapolation of LUR models to predict pollutant concentrations up to 17 years earlier and 5–10 years later has been used and validated in other studies [44–46]. Concentrations were obtained for each pollutant at each participants’ address for each year of the study.

Although PM_2.5_ and NO_2_ measures were available from fixed monitoring sites, BC was not directly measured and available. To obtain BC estimates for extrapolation, temporal NO_2_ concentration ratios were used as a proxy for temporal changes to BC, as the concentration of BC and NO_2_ were correlated (*r* = 0.7). The method to obtain these is described elsewhere [42].

For the analyses, exposures to PM_2.5_, NO_2_ and BC were considered in a continuous, time-varying manner, where the average annual pollutant concentration was allowed to vary for each year of follow-up, i.e., a one-yearly moving average, from the year of recruitment to either the outcome of interest, death, or the end of follow-up, whichever occurred first.

### Assessment of outcome

Incident lung and bladder cancer cases diagnosed between 1996 and December 2018 were ascertained by data linkage to the WA Cancer Registry, which records all cases of cancer in WA (see Supplementary Methods) [47]. The respective International Classification of Diseases for Oncology, third edition (ICD-O-3) topography codes were C34.0–34.9 for malignant lung cancer, and C67.0–67.9 for malignant bladder cancer. For those with multiple lung or bladder cancers, only the first malignant cancer was included in their respective cohorts. Participants with a diagnosis of lung and bladder cancer prior to recruitment were excluded from these analyses. We also excluded metastatic lung and bladder cancer. Participants with other cancer types were included in the study.

Two histological subtypes of lung cancer were investigated, categorised by ICD-O-3 histology codes. These were: SCC (8051–8084) and adenocarcinoma (8140–8149, 8160–8162, 8190–8221, 8260–8337, 8350–8351, 8570–8576, 8940–8941). These subtypes accounted for 50% of all lung cancers observed. Other histological groups of lung cancer (including basal cell carcinoma, other specific carcinomas, unspecified carcinomas and sarcomas and soft tissue tumours) were not studied separately, as there were too few events.

### Other variables

Data were collected at recruitment using questionnaires and health assessments, and the following baseline data were considered for analyses: age, height and weight, socioeconomic status (SES) and the self-reported variables: country of birth, smoking status, alcohol consumption, physical activity [40]. The SES was based on residential postcodes and determined through the Socio-Economic Indices For Areas, an index which was developed by the Australian Bureau of Statistics [48]. To determine which potentially confounding variables to adjust for, we developed Direct Acyclic Graphs (DAGs) on variables based upon the literature [14, 40], and used the online platform http://www.dagitty.net/ (DAGitty version 3.0, Nijmegen, NL). These are described in Supplementary Figs. S1 and S2. For lung cancer, the minimal adjustment set was participant age and SES, and for bladder cancer, the minimal adjustment set was age, SES and smoking status. Smoking status was also included in the analysis for lung cancer, as it is the strongest known risk factor for lung cancer [2]. Details of these covariates are presented in the Supplementary Methods.

### Statistical methods and data analyses

Of the 12,203 participants in the original HIMS, because our air pollution estimates were restricted to the Perth Metropolitan Area, 476 men were excluded for this reason (Supplementary Fig. S3). A further 48 participants were excluded due to missing data on smoking status (*n* = 43) and SES (*n* = 5), leaving 11,679 study participants. For the lung cancer analyses, we excluded participants with lung cancer diagnosed prior to HIMS recruitment (*n* = 62), leaving 11,617 participants for this analysis. Similarly, for bladder cancer, participants with previous diagnoses (*n* = 52) were excluded, leaving 11,627 participants for the bladder cancer analysis.

Cox proportional hazards regression analyses, with age as the timescale, were used to model the association of pollutant concentrations, on both lung and bladder cancer incidence. Separate models were performed for lung and bladder cancers, and the event of interest was the time of the first primary cancer of interest. For each respective cancer analysis, participants without the event were censored at either their date of death or at the end of the follow-up period of 31 December 2018, whichever occurred first. Mortality data were available from the WA mortality register. Both lung and bladder cancer models were adjusted for baseline area-level SES and smoking status. As there is evidence that smoking may be an effect modifier in the relationship between air pollution and lung cancer [14, 15, 22, 35], we also ran models stratifying by smoking status. In these stratified models, we provide estimates of the relationships between each pollutant and cancer outcome for each smoking status strata.

Two-pollutant models were developed for PM_2.5_, NO_2_ and BC. That is, for each pollutant (PM_2.5_, NO_2_ or BC), we also adjusted for each of the other two pollutants, in separate models. All two-pollutant models were also adjusted for baseline SES and smoking status.

The proportional hazards assumption was assessed using plots of Schoenfeld residuals, and no violations were detected. To allow for non-linearity in the relationship between each pollutant and each cancer outcome, we considered models where each pollutant was entered as a restricted cubic spline with three knots. Knot locations were determined by the recommendations in Harrell [49]. When modelling non-linear relationships, it is not appropriate to present a single hazard ratio (HR), as the HR is not constrained to be constant across the exposure range as seen when modelling a linear relationship. As we have modelled the relationship between air pollutants and incident cancers non-linearly, we report HRs and 95% confidence intervals (95% CIs) for multiple pollutant concentrations occurring within the concentration range observed in our study, when compared with a reference, also within our study range. This helps to describe how the relationship may change over the range of pollutant concentrations. Specifically, PM_2.5_ concentrations of 3, 5, and 7 µg/m^3^ are compared with a reference value of 1 µg/m^3^, NO_2_ concentrations of 10, 15, and 25 µg/m^3^ are compared with a reference value of 5 µg/m^3^, and BC concentrations of 0.8 × 10^−5^, 1.2 × 10^−5^, and 1.6 × 10^−5^ m^−1^ are compared with a reference value of 0.5 × 10^−5^ m^−1^.

Five sets of sensitivity analyses were conducted. The first was assigning a baseline pollutant exposure to all participants. This was the average concentration of each pollutant during the participant’s recruitment year. Current studies have used baseline concentrations to estimate participant exposures to ambient air pollutants [14, 38, 43]. This analysis would enable us to compare our findings with other studies that have used similar exposure measures, as well as with comparing with the use of time-varying measures in our study.

When using age as the timescale, comparisons are made between participants at specific ages, as opposed to specific calendar years of exposure. As such, we conducted a sensitivity analysis which included an interaction between birth cohort (birth years 1914–1924 and 1925–1932) and each air pollutant to see whether the impact of air pollution on lung and bladder cancer differed for those born at different times.

The third set of sensitivity analyses excluded participants who had a lung or bladder cancer diagnosis in the first two years of follow-up, in addition to those who were excluded due to diagnoses prior to recruitment. This factored in lung or bladder cancer cases at baseline that may have had delayed diagnosis or registration to the WA Cancer Registry.

Exposure estimates were based on residential addresses at the time of recruitment, thus assuming that all participants had lived at the same residential address the whole time, and as such, a fourth set of analyses was done to exclude participants who had moved addresses (‘movers’) during the follow-up period. Participants who had moved addresses from the baseline during the study were recorded in the HIMS dataset.

Finally, we also included all available covariates from the DAGs in the primary analyses to assess the robustness of our models.

All analyses were performed using Stata software package 17.0 (StataCorp, College Station, TX).

## Results

### Study population characteristics

Baseline demographic and lifestyle characteristics of the study participants are presented in Table 1. The mean age at baseline was 72.1 (SD ± 4.4) years old. Most participants had high area-level SES (64%) and were former smokers (59.6%). Most of the former smokers had quit more than 10 years prior to recruitment.

**Table 1:** Baseline demographic and lifestyle characteristics of men, HIMS 1996–1999, in Perth, Western Australia.

Demographic and lifestyle covariates	Baseline (1996–1999) *n* = 11,679^a^
Age, mean (SD)	72.1 (±4.4)
Smoking status, *n*%	
Never smoker	3430 (29.4%)
Former smokers (quit smoking ≥10 years before recruitment date)	5624 (48.1%)
Former smokers (quit smoking <10 years before recruitment date)	1333 (11.4%)
Current smoker	1292 (11.1%)
Region of birth^a^, *n*%	
Australia	6412 (54.9%)
Europe	4372 (37.5%)
Other regions^b^	892 (7.6%)
Education^a^, *n*%	
Completed university	1868 (16.0%)
Completed high school	2824 (24.2%)
Did not complete high school	6980 (59.8%)
SES status, *n*%	
High	7503 (64.2%)
Middle	3689 (31.6%)
Low	487 (4.2%)
Physical activity (h/week), *n*%	
Active (vigorous activity and non-vigorous physical activity ≥150 min/week)	7186 (61.5%)
Inactive (vigorous and non-vigorous physical activity <150 min/week)	4493 (38.5%)
Job status, *n*%	
Tradepersons, labourers and related workers	5123 (43.9%)
Non-trade-related work, non-labourers and all other work types	6556 (56.1%)
Movers vs non-movers status^c^, *n*%	
Movers	1146 (9.8%)
Non-movers	10,533 (90.2%)

^a^Numbers do not always add to 11,679 due to missing data: region of birth (*n* = 3), education status (*n* = 7).

^b^Other regions include: South-east Asia 1.9%, Other Asia 3.3%, Africa 1.4%, Other Oceania 0.5%, and America (0.5%).

^c^Movers are participants who did move residential addresses during follow-up, non-movers are participants who did not move residential addresses during follow-up.

### Air pollutant concentrations of study population

Average concentrations in the starting year of the study (1996) were: 5.65 µg/m^3^ for PM_2.5_, 15.83 µg/m^3^ for NO_2_, and 1.06 × 10^−5^ m^−1^ for BC. Average concentrations at the end of the study (2018) were: 4.35 µg/m^3^ for PM_2.5_, 11.20 µg/m^3^ for NO_2_, and 0.81 × 10^−5^ m^−1^ for BC. Further distributional information is described in Supplementary Table S1. Pairwise correlations between the three pollutants at recruitment all had Spearman’s rho (*r*) <0.7 (Supplementary Table S2). Participants with cancer events had slightly higher mean concentrations of PM_2.5_, NO_2_ and BC, compared to those who did not have the respective cancer event at recruitment (Table 2).

**Table 2:** Ambient air pollutants concentrations for the HIMS cohort at recruitment, stratified by lung or bladder cancer, in Perth, Western Australia.

Pollutant type (mean, SD)	Ambient air pollutant concentrations stratified by event and cohort		
	Cancer event	No event	Total cohort
*Lung cancer cohort (n* = *11,617)*			
	*n* = 692	*n* = 10,925	*n* = 11,617
PM_2.5_ (µg/m^3^)	5.28 (±1.56)	5.04 (±1.69)	5.06 (±1.68)
NO_2_ (µg/m^3^)	13.62 (±4.02)	13.41 (±4.10)	13.42 (±4.09)
BC (×10^−5^ m^−1^)	0.99 (±0.25)	0.97 (±0.27)	0.97 (±0.27)
*Bladder cancer cohort (n* = *11,627)*			
	*n* = 224	*n* = 11,403	*n* = 11,627
PM_2.5_ (µg/m^3^)	5.06 (±1.62)	5.06 (±1.69)	5.06 (±1.68)
NO_2_ (µg/m^3^)	13.66 (±4.31)	13.42 (±4.09)	13.42 (±4.09)
BC (×10^−5^ m^−1^)	0.98 (±0.27)	0.97 (±0.27)	0.97 (±0.27)

### Associations between ambient air pollutants and lung cancer

There were 692 (6.0%) lung cancer events identified during the study period, with a total follow-up time of 158,479 person-years.

Positive associations between the pollutants and lung cancer were observed in both crude and adjusted models (Table 3). In the adjusted single-pollutant model, HRs for PM_2.5_ and BC, but not NO_2_, were mostly statistically significant. In the two-pollutant models, the results for PM_2.5_ were attenuated, and lost statistical significance, when controlling, separately, for NO_2_ and BC. The results for BC were attenuated when controlling for PM_2.5_ but not for NO_2_, but stayed statistically significant.

**Table 3:** Associations between ambient air pollutant concentrations, and incident lung and bladder cancer in the HIMS cohort.

Air pollutant concentration^a^	Single-pollutant models		Two-pollutant models^b^		
	Unadjusted	Adjusted^b^	Adjusted for NO_2_	Adjusted for BC	Adjusted for PM_2.5_
	HR (95% CI)	HR (95% CI)	HR (95% CI)	HR (95% CI)	HR (95% CI)
*Total lung cancer cohort (n* = *692), total person-years* = *158,479*					
PM_2.5_					
3 vs 1	1.38 (1.10, 1.71)	1.25 (1.00, 1.56)	1.23 (0.97, 1.54)	1.15 (0.91, 1.45)	N/A
5 vs 1	1.76 (1.26, 2.46)	1.45 (1.03, 2.03)	1.39 (0.98, 1.99)	1.25 (0.87, 1.80)	
7 vs 1	1.95 (1.42, 2.68)	1.43 (1.02, 2.00)	1.36 (0.95, 1.95)	1.23 (0.85, 1.78)	
NO_2_					
10 vs 5	1.15 (0.93, 1.42)	1.08 (0.88, 1.33)	N/A	0.82 (0.63, 1.08)	1.02 (0.82, 1.26)
15 vs 5	1.28 (0.94, 1.74)	1.16 (0.85, 1.57)		0.76 (0.50, 1.16)	1.06 (0.77, 1.45)
25 vs 5	1.44 (1.02, 2.05)	1.33 (0.94, 1.89)		0.95 (0.59, 1.54)	1.19 (0.82, 1.72)
BC					
0.8 vs 0.5	1.38 (1.15, 1.67)	1.30 (1.07, 1.57)	1.44 (1.12, 1.84)	N/A	1.24 (1.02, 1.52)
1.2 vs 0.5	1.59 (1.24, 2.04)	1.45 (1.12, 1.86)	1.63 (1.14, 2.32)		1.35 (1.03, 1.78)
1.6 vs 0.5	1.47 (1.08, 2.02)	1.35 (0.98, 1.86)	1.41 (0.91, 2.18)		1.25 (0.88, 1.77)
*Total bladder cancer cohort (n* = *224), total person-years* = *158,440*					
PM_2.5_					
3 vs 1	1.20 (0.84, 1.70)	1.18 (0.83, 1.70)	1.17 (0.80, 1.70)	1.14 (0.77, 1.67)	N/A
5 vs 1	1.27 (0.75, 2.14)	1.25 (0.73, 2.14)	1.20 (0.68, 2.14)	1.16 (0.64, 2.09)	
7 vs 1	1.05 (0.62, 1.79)	1.03 (0.59, 1.82)	0.97 (0.53, 1.78)	0.93 (0.49, 1.75)	
NO_2_					
10 vs 5	0.99 (0.70, 1.41)	0.98 (0.69, 1.39)	N/A	0.90 (0.56, 1.43)	0.95 (0.66, 1.37)
15 vs 5	1.04 (0.62, 1.74)	1.01 (0.61, 1.69)		0.88 (0.43, 1.80)	0.98 (0.57, 1.68)
25 vs 5	1.38 (0.76, 2.51)	1.32 (0.73, 2.41)		1.16 (0.50, 2.70)	1.32 (0.69, 2.50)
BC					
0.8 vs 0.5	1.08 (0.80, 1.45)	1.06 (0.79, 1.43)	1.11 (0.75, 1.66)	N/A	1.05 (0.76, 1.44)
1.2 vs 0.5	1.18 (0.79, 1.75)	1.15 (0.77, 1.71)	1.17 (0.66, 2.07)		1.14 (0.73, 1.78)
1.6 vs 0.5	1.28 (0.74, 2.20)	1.24 (0.72, 2.15)	1.14 (0.54, 2.43)		1.28 (0.70, 2.34)

Single- and two-pollutant-adjusted hazard ratios were estimated across four models.

*N/A* not applicable.

^a^Hazard ratios considered reference values of: 1 µg/m^3^ for PM_2.5_, 5 µg/m^3^ for NO_2_, and 0.5 × 10^−5^ m^−1^ for BC. Age is used as the timescale.

^b^Adjusted for smoking status and socioeconomic status.

### Ambient air pollution and lung cancer histological subtypes

In the lung cancer cohort, there were 138 (1.2%) SCCs and 201 (1.7%) adenocarcinomas of the lung reported during follow-up (Table 4). Concentrations of PM_2.5_ and BC of participants were positively associated with both SCC and adenocarcinoma, across the unadjusted and adjusted single-pollutant models, although these were not significant. The HRs in the respective two-pollutant models were further attenuated, except for BC when controlled for NO_2_. Concentrations of NO_2_ were positively associated with SCC, but not ADC in the single-pollutant models. No associations between incident lung cancer and NO_2_ were observed across all two-pollutant models.

**Table 4:** Associations between ambient air pollutant concentrations, and incident lung cancer by histological subtypes, in the HIMS cohort.

Air pollutant concentration^a^	Single-pollutant models		Two-pollutant models^b^		
	Unadjusted	Adjusted^b^	Adjusted for NO_2_	Adjusted for BC	Adjusted for PM_2.5_
	HR (95% CI)	HR (95% CI)	HR (95% CI)	HR (95% CI)	HR (95% CI)
*Squamous cell carcinoma (n* = *138), total person-years* = *159,190*					
PM_2.5_					
3 vs 1	1.34 (0.81, 2.20)	1.18 (0.71, 1.94)	1.15 (0.68, 1.94)	1.01 (0.59, 1.72)	N/A
5 vs 1	1.78 (0.83, 3.81)	1.36 (0.63, 2.95)	1.30 (0.58, 2.93)	1.05 (0.46, 2.41)	
7 vs 1	2.36 (1.15, 4.84)	1.53 (0.72, 3.28)	1.42 (0.63, 3.20)	1.16 (0.50, 2.68)	
NO_2_					
10 vs 5	1.06 (0.67, 1.68)	0.98 (0.62, 1.55)	N/A	0.61 (0.33, 1.11)	0.93 (0.58, 1.50)
15 vs 5	1.18 (0.59, 2.33)	1.04 (0.53, 2.05)		0.50 (0.20, 1.25)	0.96 (0.47, 1.94)
25 vs 5	1.69 (0.80, 3.58)	1.55 (0.73, 3.28)		0.83 (0.29, 2.36)	1.37 (0.62, 3.04)
BC					
0.8 vs 0.5	1.59 (1.01, 2.50)	1.47 (0.93, 2.32)	1.94 (1.07, 3.51)	N/A	1.45 (0.89, 2.34)
1.2 vs 0.5	2.00 (1.10, 3.66)	1.79 (0.97, 3.30)	2.49 (1.07, 5.79)		1.72 (0.89, 3.34)
1.6 vs 0.5	1.88 (0.92, 3.86)	1.71 (0.83, 3.53)	1.98 (0.73, 5.39)		1.62 (0.74, 3.55)
*Adenocarcinoma (n* = *201), total person-years* = *159,113*					
PM_2.5_					
3 vs 1	1.15 (0.79, 1.67)	1.07 (0.74, 1.57)	1.11 (0.75, 1.65)	0.99 (0.66, 1.49)	N/A
5 vs 1	1.23 (0.71, 2.14)	1.04 (0.59, 1.84)	1.11 (0.61, 2.02)	0.91 (0.49, 1.69)	
7 vs 1	1.12 (0.64, 1.96)	0.81 (0.45, 1.48)	0.88 (0.46, 1.68)	0.71 (0.36, 1.38)	
NO_2_					
10 vs 5	1.03 (0.71, 1.50)	0.97 (0.67, 1.41)	N/A	0.72 (0.44, 1.18)	0.97 (0.66, 1.42)
15 vs 5	1.00 (0.58, 1.72)	0.91 (0.53, 1.56)		0.56 (0.27, 1.17)	0.91 (0.52, 1.60)
25 vs 5	0.78 (0.40, 1.54)	0.72 (0.36, 1.42)		0.44 (0.18, 1.09)	0.74 (0.36, 1.53)
BC					
0.8 vs 0.5	1.26 (0.90, 1.76)	1.18 (0.84, 1.66)	1.47 (0.94, 2.28)	N/A	1.22 (0.85, 1.75)
1.2 vs 0.5	1.33 (0.85, 2.08)	1.21 (0.77, 1.90)	1.80 (0.96, 3.38)		1.31 (0.80, 2.16)
1.6 vs 0.5	1.17 (0.65, 2.12)	1.07 (0.59, 1.93)	1.75 (0.79, 3.88)		1.22 (0.64, 2.34)

Single- and two-pollutant-adjusted hazard ratios were estimated across four models.

*N/A* not applicable.

^a^Hazard ratios considered reference values of: 1 µg/m^3^ for PM_2.5_, 5 µg/m^3^ for NO_2_, and 0.5 × 10^−5^ m^−1^ for BC. Age is used as the timescale.

^b^Adjusted for smoking status and socioeconomic status.

### Associations between ambient air pollutants and bladder cancer

There were 224 incident bladder cancer events (1.9%), during 158,440 person-years of follow-up. Concentrations of PM_2.5_ and BC were positively associated with bladder cancer in their respective single and two-pollutant models, although these were not statistically significant (Table 3). A weak positive association was observed between NO_2_ and bladder cancer in single-pollutant models; however, this was attenuated when adjusted for other co-pollutants.

**Fig. 1:**
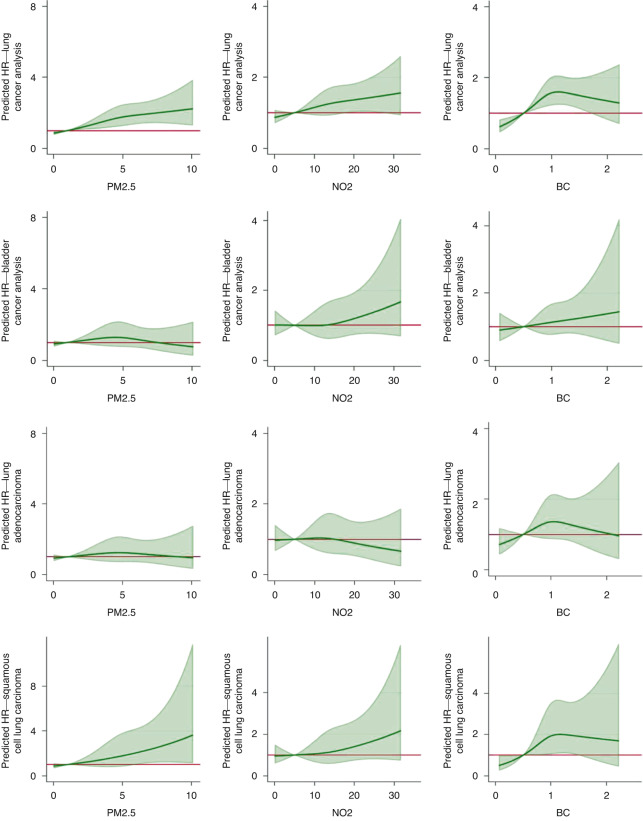
Graphs showing 3-knot restricted cubic spline regression demonstrating non-linear associations between ambient air pollutants and incident lung cancer, bladder cancer, and histological subtypes of lung cancer (adenocarcinoma and squamous cell carcinoma), in the HIMS cohort for the single-pollutant unadjusted models. i) Lung cancer: PM_2.5_. ii) Lung cancer: NO_2_. iii) Lung cancer: BC. iv) Bladder cancer: PM_2.5_. v) Bladder cancer: NO_2_. vi) Bladder cancer: BC. vii) ADC: PM_2.5_. viii) ADC: NO_2_. ix) ADC: BC. x) SCC: PM_2.5_. xi) SCC: NO_2_. xii) SCC: BC.

We observed evidence of a non-linear relationship between pollutants and both lung and bladder cancer (Fig. 1).

### Stratification by smoking status

When stratified by smoking status, positive associations were observed between all pollutants and lung cancer for the former smokers’ group who had quit smoking ≥10 years ago in the single-pollutant models, but not consistently in other smoking groups (Supplementary Table S3). Bladder cancer estimates were relatively consistent across the different smoking cohorts.

### Sensitivity analyses

#### Baseline pollutant exposure

For lung cancer, the HRs associated with baseline concentrations of PM_2.5_ and BC remained mostly significant in the crude and adjusted single-pollutant models (Supplementary Table S4). A positive, but non-significant, association was observed between incident lung cancer and NO_2_ in the single-pollutant models. Positive associations between baseline exposures of PM_2.5_ and BC and incident bladder cancer were also observed, but not with NO_2._ In the lung cancer histological subtype analysis, the incidence of SCC was positively associated with baseline concentrations of all pollutants in the crude and adjusted single-pollutant models; however, the confidence intervals were very wide (Supplementary Table S5). Positive associations were also observed between incident lung adenocarcinoma with PM_2.5_ and BC, but not with NO_2_. Evidence of a non-linear relationship was observed between ambient air pollutants, and both lung and bladder cancer (Supplementary Fig. S4).

#### Birth cohort

For both lung and bladder cancer analyses, no significant interactions were observed between ambient air pollutants and the birth cohorts (all *p* values > 0.09).

#### Two-year cancer exclusion

The overall findings were similar when participants with incident lung or bladder cancer in the first 2 years of follow-up were excluded (Supplementary Table S6).

#### Excluding movers

The associations between PM_2.5_, NO_2_ and BC and both lung and bladder cancer remained similar after excluding participants who had moved residential addresses during the follow-up period (Supplementary Table S7).

#### Inclusion of all covariates in the primary analyses

We also did not observe significant changes in associations when other available covariates were added to the models (Supplementary Table S8).

## Discussion

We observed positive associations between follow-up exposures to PM_2.5_, NO_2_ and BC, and both incident lung and bladder cancer. The concentrations of PM_2.5_ and NO_2_ in this study were low and, on average, below Australian air quality standards (current annual average thresholds are 8 µg/m^3^ and 15 ppb (≈28 µg/m^3^), respectively [39]), and lower than has been reported in other ‘low-pollution’ studies [14–19, 35, 36].

The range of exposure concentrations for each pollutant reported in our study was narrow. The annual average concentrations (and range) during follow-up for: PM_2.5_ was 4.63 µg/m^3^ (0.01–9.20 µg/m^3^), NO_2_ was 13.59 µg/m^3^ (0.82–29.02 µg/m^3^), and BC was 0.98 × 0.5 × 10^−5^ m^−1^ (0.08–2.03 × 10^−5^ m^−1^). Our findings support the notion that there may not be a threshold concentration for the carcinogenic effects of ambient air pollution.

### Lung cancer and PM_2.5_

The associations between PM_2.5_ and lung cancer were consistent with recent studies conducted in the USA [15, 35], Canada [17–19], and Europe [14, 50], where an increase in lung cancer risk was observed with relatively low concentrations of PM_2.5_ exposures. For example, the ELAPSE study [14], a pooled cohort of seven countries in Europe, reported a median annual concentration of 15.4 µg/m^3^, and a pooled-HR of 1.13 (95% CI: 1.05–1.23) per 5 µg/m^3^ increase in PM_2.5_. Studies in Canada with lower exposure concentrations reported HRs of 1.02 (95% CI: 1.01–1.05) per 5.3 µg/m^3^ [19], 1.16 (95% CI: 1.07–1.25) per 10 µg/m^3^ [17], and 1.34 (95% CI: 1.10–1.65) per 10 µg/m^3^ [18], where average respective PM_2.5_ concentrations were 7.68 µg/m^3^ (mean), 7.4 µg/m^3^ (mean), and 9.1 µg/m^3^ (median). On the other hand, the Biobank study [50] in the United Kingdom (UK) reported a high HR of 1.63 (95% CI: 1.33–2.01) per 5 µg/m^3^ increase in PM_2.5_ with mean PM_2.5_ concentration of 10 µg/m^3^. Recent studies (with a wider exposure range) have suggested that the relative risk of lung cancer (with each incremental increase in pollutant) may be greater at lower concentrations of PM_2.5_, compared to higher concentrations [14, 17, 19]. Because of the narrow exposure range in our study, we could not investigate this further.

### Lung cancer and NO_2_ and BC

The association between ambient NO_2_ and lung cancer has regularly been observed [16, 19, 23–26, 29–32, 50, 51], although not in all studies [27, 28]. In regions with low NO_2_, significant associations with lung cancer have been found in studies conducted in the Netherlands [23–25], the UK [50], the USA [26], and Canada [16, 19]. The average concentrations of NO_2_ reported in these studies were below European Air Quality Standards of <40 µg/m^3^ [52]. In our study, the NO_2_ concentrations were below the Australian standard of 28 µg/m^3^. We observed non-significant associations between NO_2_ and lung cancer, which was a similar finding to a pooled analysis of European cohorts in relatively low-pollution areas (HR = 1.02, 95% CI = 0.97–1.07) [14].

We observed significant associations between BC and lung cancer in all models. There is currently a paucity of studies on BC and lung cancer. We identified three studies and one pooled analysis that investigated associations between BC and lung cancer [14, 23, 25, 33]. Two studies found significant associations with lung cancer; [23, 25] the multi-centre European ELAPSE Study did not find an association between lung cancer and BC [14], and the French Gazel Cohort Study [33] reported non-statistically significant associations between lung cancer and BC. Given the limited studies, further research is needed to better understand potential associations.

In 2012, the IARC declared diesel engine exhaust as a Group 1 human carcinogen [53]. Ambient BC is a common proxy for diesel [54] and, therefore, our findings may reflect the association between diesel engine exhaust and lung cancer. In Australia, the proportion of cars that are diesel powered is growing steadily and accounts for over 26% of all motor vehicles in this country [55]. Despite efforts to reduce total emissions in ambient air, there is a growing trend towards driving diesel-powered sports utility and utility vehicles, leading to greater production of tail-pipe pollutants [56].

Whilst positive associations were observed between both NO_2_ and BC and lung cancer, when analysed together, a positive, but non-significant association, with lung cancer remained with BC but not with NO_2_. This suggests that the association of NO_2_ with lung cancer is in part reflecting the effects of BC. The HRs were stronger when NO_2_ was included in the model with BC, suggesting there may be additive effects. As both NO_2_ and BC are produced from traffic-related pollution, they are often present together [33].

### Air pollutants and lung cancer histological subtypes

The relationship between histological subtypes of lung cancer and ambient air pollution remains unclear. Positive associations were observed between BC and both SCC and adenocarcinoma, across both single and two-pollutant models. Positive, but non-significant, associations were also observed for PM_2.5_ and both histological lung cancer subtypes in the single-pollutant models. The effects were attenuated in the two-pollutant models, particularly for lung adenocarcinoma. Concentrations of NO_2_ were positively associated with increasing risk of SCC; however, effects were also attenuated after controlling for other pollutants. No associations were observed for NO_2_ with adenocarcinoma.

Our findings contrast the results from previous studies, where stronger associations between PM_2.5_ and adenocarcinoma have been observed, compared with other lung cancer subtypes [18, 43]. The ELAPSE study also observed associations between PM_2.5_ and adenocarcinoma, but not with SCC [14]. However, no associations were observed between NO_2_ or BC, with either adenocarcinoma and SCC [14]. Hart et al. [23]. also reported no associations for between different histological subtypes of lung cancer, and either NO_2_ or black smoke. These studies used single-pollutant models and did not adjust for co-pollutants. The risk of incident SCC (and not adenocarcinoma) was not found to be associated with PM_2.5_, NO_2_ and BC in previous studies.

### Bladder cancer and PM_2.5_, NO_2_ and BC

There was a positive association between PM_2.5_ and bladder cancer, although this was not statistically significant. This is consistent with the multi-centre European study, the ELAPSE Study [36], where a positive but non-significant association was identified. Positive associations between ambient air pollution and bladder cancer were also described in the report by IARC in 2013 [10] and in a more recent study in the USA [27]. However, no associations were observed for other studies conducted in Spain [37], the USA [35], and another pooled multi-cohort study in Europe [38].

Exposures to NO_2_ were not associated with bladder cancer, which is consistent with existing literature. No associations with bladder cancer were reported in studies in Spain [37], the USA [27], and a multi-centre European study [38]. However, a positive, albeit non-significant, association was observed with BC across all pollutant models. This differed from the ELAPSE Study [36], where no association between BC and bladder cancer was reported. We observed that NO_2_ and BC were not independent of each other, and the presence of both in the co-pollutant analysis resulted in attenuation of their respective effects on bladder cancer.

### Baseline pollutant exposures

Associations using baseline exposures for ambient air pollutants were similar to the time-varying exposures, for both lung and bladder cancer analyses. The pattern of associations between baseline concentrations of PM_2.5_, NO_2_ and BC and both respective cancers remained consistent in the sensitivity analyses (Supplementary Tables S4 and S5). The use of pollutant exposures estimated at residential home addresses at the time of recruitment as a measure of long-term pollutant exposure is a common method used in the literature to examine risk of chronic health effects [14, 38, 43]. However, assigning time-varying exposures provides a more precise assignment and enables a greater detection in the effect measure, which may be more important in low-level air pollution studies. A Canadian study investigating low-level ambient air pollution and mortality also observed differences in these effect measures, and suggested that time-varying estimates for these studies are important, particularly for pollutants with high spatial resolution [16]. However, the ELAPSE Study observed minimal difference in effect measures with different exposure metrics including baseline, annual average, and time-varying concentrations in their sensitivity analyses [14].

### Stratified by smoking status

We observed modest effect modification by smoking status in the lung cancer analysis, and this has also been observed in the literature [14, 15, 22, 35]. Positive associations between lung cancer and ambient air pollutants were observed in the group that had quit smoking ≥10 years ago, but not the never-smoker group. Positive associations have been observed in studies between lung cancer and PM_2.5_ in never-smokers [14, 15]. Another study observed positive associations when analysis was restricted to participants who had never smoked, or had quit ≥10 years ago [22]. A recent meta-analysis also found a higher risk of lung cancer with PM_2.5_ in never and former smokers, compared to current smokers; however, the results were imprecise [12]. Another study found PM_2.5_-associated lung cancer mortality was higher in the never-smokers cohort (HR = 1.73, 95% CI = 1.20–2.49) compared to the full cohort (HR = 1.13, 95% CI = 1.00–1.26) [35].

Smoking status did not modify the effect of ambient air pollutants and bladder cancer in our study. Our findings were similar to other studies, where a non-significant interaction between smoking status [36] and pack-years smoked [37] was observed with ambient air pollutants.

### Strengths and limitations

Our study had several strengths. The follow-up time of 22 years was suitable for lung and bladder cancer, which both have long latency periods. Analysing data from an older-aged cohort is a consideration of a population particularly vulnerable to the health effects of hazardous air pollution. The HIMS is linked to the WADLS and follow-up data for all participants were available through data linkage, including to state hospital records and mortality registry. Reporting cancer diagnoses to the WA Cancer Registry is also a mandatory requirement for WA health practitioners [47]. The WADLS has stringent checking procedures, with low rates of errors in their datasets [57]. Tracking all participants reduced the potential for attrition bias that may occur from loss to follow-up.

The validated LUR models had good performance, and could explain variability in PM_2.5_, NO_2_ and BC concentrations in Perth by approximately 67%, 69% and 85%, respectively [41, 42]. However, whilst LUR models have been used and validated for back- and forward-extrapolation in previous studies, we did not have validation data to justify the extrapolation of our models for Perth (WA).

The HIMS cohort only included older, mainly Caucasian men, limiting generalisability. Many studies in the literature have used large-scale cohorts (such as populations across multiple countries and sites [14, 50]), and have drawn from national or state-wide administrative and health surveillance databases [17–19, 35]. These studies include both middle and older-aged adults, and both males and females, resulting in a greater heterogeneity of participants. Female-only studies have also been conducted, such as participants recruited from the Canadian National Breast Screening Study [18]. In contrast, the HIMS cohort was originally recruited for a randomised screening trial for Abdominal Aortic Aneurysms [40], and included older-aged men from a single city only.

Although we reported a large number of lung cancer events, the case counts for bladder cancer and histological subtypes of lung cancer were relatively fewer. This could limit the statistical power of these analyses. Whilst our sample size was relatively small compared to other studies, our cohort was a subset of the population most vulnerable to lung (and bladder) cancers. Evaluating lesser-known cancer risk factors such as air pollution was, therefore, suitable and relevant for our cohort.

Pollution estimates were from residential addresses and did not consider where they spent their time. Personal exposure to air pollution is dependent on the day-to-day microenvironments of the individual, which include both indoor and outdoor exposures at their home [58].

Other residual confounding for lung cancer risk may not have been considered. For example, the UK Biobank Study observed that genetic factors could increase the effect of ambient air pollution on lung cancer by 50% [50]. Genetic factor analyses were outside the study scope. Finally, confounding was determined at recruitment; hence, lifestyle changes (such as smoking status) during follow-up were not considered.

## Conclusion

In conclusion, we found an increased risk of lung and possible increase of bladder cancer in a cohort of older men in Perth, WA, residing in areas where ambient air pollution concentrations are typically considered very low. This adds to the increasing evidence that negative health effects are being identified at low concentrations of air pollution. Further studies are needed to understand the carcinogenic potential of ambient air pollution at low concentrations.

### Supplementary information

Supplementary Information

## Data Availability

Data were obtained from the HIMS, and HIMAQs, and the WADLS. Access to these datasets requires approval from these groups.
