# Supplementary material for: Exposure to low-level ambient air pollution and the relationship with lung and bladder cancer in older men, in Perth, Western Australia
Source: Br J Cancer. 2023 Sep 8;129(9):1500–9. doi: 10.1038/s41416-023-02411-x (PMC10628106; doi:10.1038/s41416-023-02411-x)
Supplement: Supplementary file 1 — Supplementary Information [file 41416_2023_2411_MOESM1_ESM.docx]

Supplementary Information

### Table of contents:

1. Supplementary Methods

2. Supplementary Tables

Table S1: Ambient air pollution concentrations – additional distribution information
Table S2: Spearman correlation coefficient table of baseline pollutant concentrations
Table S3: Lung and bladder cancer analysis - stratified by smoking status

Table S4: Sensitivity analysis using pollutant baseline concentrations assigned to participants at recruitment – lung cancer and bladder cancer analysis

Table S5: Sensitivity analysis using pollutant baseline concentrations assigned to participants at recruitment – lung histological subtype analysis

Table S6: Sensitivity analysis excluding lung and bladder cancer incidence during the first 2 years of follow-up in the HIMS cohort

Table S7: Sensitivity analysis excluding movers in the HIMS cohort

Table S8: Ambient air pollution and lung and bladder cancer analysis – additional models to include all area-level SES variables, and all other available covariates

3. Supplementary Figures

Figure S1: Directed acyclic graph describing association between confounding factors and lung cancer

Figure S2: Directed acyclic graph describing association between confounding factors and bladder cancer

Figure S3: Flowchart of the HIMS recruitment and selection process

Figure S4: Graphs of 3-knot restricted cubic spline regression using air pollutant concentrations at recruitment (baseline)

4. References

### Supplementary Methods

The sections below will expand on the following topics:

i) The Western Australia Data Linkage System and specific cancer outcomes

ii) Covariates in the final model: socioeconomic status and smoking status

**i) The Western Australia Data Linkage System and the Western Australia Cancer Registry (specific cancer outcomes)**

The Western Australia Data Linkage System (WADLS) commenced in 1995.^1^ It consists of more than 50 data collections from Western Australia.^1^ The WADLS is linked to the Western Australia Cancer Registry (WACR), and records are updated monthly.^1^ Diagnosis of cancer to the WACR is a statutory reporting requirement under the Health (Notification of Cancer) Regulations 2011.^2^ Cancer data is reported by pathologists (including haematologists) and radiation oncologists, and include a written report and/or pathology reports submitted to the registry.^2^ As diagnosing or treating physicians are providing the reports directly, the risk of reporting error of cancer and histological subtypes is minimal. Governance, including data validation, is addressed in the Data Quality Statement for the WA Cancer Register.^2^

**ii) Covariates in the final model: socioeconomic status (SES) and smoking status**

Several covariates that were used by current studies were available in our study. However, we only included the minimum adjusted set required for confounding in our models, identified using Directed Acyclic Graphs (DAGs). Although smoking was not part of the minimum adjusted set identified for confounding the lung cancer DAG, we still included it in the lung cancer models.

SES is a categorical variable, based on the Socio-Economic Indexes for Areas (SEIFA Index) from the Australian Bureau of Statistics. Each Index focuses on a different subset of Census variables and aspects of socioeconomic advantage and disadvantage within an area. Of the various SEIFA Index available, we selected The Index of Relative Socio-economic Advantage and Disadvantage (IRSAD), as this was most relevant for our study. The IRSAD consists of 10 deciles, which we regrouped to three groups: Low SES (decile 1-3), Medium SES (decile 4-7) and High SES (decile 8-10).

Smoking status is also a categorical variable consisting of: never smoker, quit smoking > 10 years ago, quit smoking < 10 years ago, and current smoker.

### ****Table S1. Additional distributional information for ambient air pollutant exposures to the HIMS participants, stratified by lung and bladder cancer cohorts****

Additional distributional information on ambient air pollutants exposures are described for both lung and bladder cancer cohorts. These include median, range, and interquartile range (IQR). We present two exposure metrics: the time-varying concentrations (used for the primary analysis), and pollutant concentrations assigned to participants at recruitment.

| **Pollutant exposure measure^a^** | **Lung cancer cohort (n=11 617)** | | **Bladder cancer cohort (n=11 627)** | | |
| --- | --- | --- | --- | --- | --- |
|  | **Median (range)** | **IQR** | **Median (range)** | **IQR** | |
| PM_2.5_ |  | |  |  |  |
| Time-varying | 4.80 (<LoD-10.10) | 2.11 | 4.80 (<LoD-10.10) | 2.11 | |
| Recruitment | 5.35 (<LoD-10.02) | 2.24 | 5.35 (<LoD-10.02) | 2.24 | |
| NO_2_ |  | |  |  |  |
| Time-varying | 12.94 (<LoD-31.72) | 5.93 | 12.94 (<LoD-31.72) | 5.93 | |
| Recruitment | 13.18 (<LoD-27.24) | 5.56 | 13.18 (<LoD-27.24) | 5.57 | |
| BC |  | |  |  |  |
| Time-varying | 0.95 (0.07-2.22) | 0.38 | 0.95 (0.07-2.22) | 0.38 | |
| Recruitment | 0.97 (0.08-1.90) | 0.36 | 0.97 (0.08-1.90) | 0.36 | |

*^a^Concentrations expressed are: PM_2.5_ and NO_2_ = µg/m^3^, and BC = 0.5x10^-5^m^-1^*

### ****Table S2. Spearman correlation coefficient table of baseline pollutant concentrations, stratified by lung cancer and bladder cancer cohorts****

Correlation between ambient air pollutants was determined by Spearman correlation coefficients, using the pollutant concentrations at recruitment for the HIMS participants. Spearman’s rho were similar for both lung and bladder cancer cohorts.

| **Pollutant combination** | **Spearman’s rho** |  |
| --- | --- | --- |
| ***Lung cancer cohort (n=11 617)*** | | |
| PM_2.5_ and NO_2_ | 0.32 | |
| NO_2_ and BC | 0.66 | |
| BC and PM_2.5_ | 0.43 | |
| ***Bladder cancer cohort (n=11 627)*** | | |
| PM_2.5_ and NO_2_ | 0.32 | |
| NO_2_ and BC | 0.66 | |
| BC and PM_2.5_ | 0.43 | |

###

### Table S3. Incident lung and bladder cancer stratified by smoking status in the HIMS cohort

The table below shows associations between ambient air pollutant concentrations, and incident lung and bladder cancer in the HIMS cohort. Single pollutant adjusted hazard ratios were stratified across all four smoking status groups: never smoker, former smoker (quit > 10 years before recruitment date), former smoker (quit < 10 years before recruitment date), and current smoker. Models have been adjusted for socioeconomic status.

| **Air pollutant**  **concentration^a^** | **Lung cancer (N=11 617)** | | | | | | | | **Bladder cancer (N= 11 627)** | | | | | | |
| --- | --- | --- | --- | --- | --- | --- | --- | --- | --- | --- | --- | --- | --- | --- | --- |
|  | **Unadjusted** | | | **Adjusted** | | | | | **Unadjusted** | | | | | **Adjusted** | |
|  | **HR (95% CI)** | | | **HR (95% CI)** | | | | | **HR (95% CI)** | | | | | **HR (95% CI)** | |
| **Never smoker** | | **n = 3425** | | | | | | **n = 3425** | | | | | | |  |
| PM_2.5_ (µg/m^3^) |  | | | | |  | | | | | | |  | | |
| 3 vs 1 | 0.71 (0.36, 1.37) | | | 0.70 (0.36, 1.39) | | | 1.14 (0.47, 2.76) | | | | | 1.17 (0.48, 2.88) | | | |
| 5 vs 1 | 0.65 (0.25, 1.65) | | | 0.63 (0.23, 1.67) | | | 1.49 (0.41, 5.40) | | | | | 1.61 (0.43, 6.06) | | | |
| 7 vs 1 | 0.94 (0.34, 2.57) | | | 0.85 (0.28, 2.53) | | | 2.48 (0.72, 8.50) | | | | 2.94 (0.82, 10.62) | | | | |
| NO_2_ (µg/m^3^) |  | | |  | | | | |  | | | | |  | |
| 10 vs 5 | 0.97 (0.46, 2.03) | | | 0.96 (0.46, 2.03) | | | 1.43 (0.58, 3.49) | | | | 1.44 (0.59, 3.51) | | | | |
| 15 vs 5 | 1.00 (0.35, 2.85) | | | 0.99 (0.35, 2.84) | | | 1.70 (0.48, 6.05) | | | | 1.71 (0.48, 6.08) | | | | |
| 25 vs 5 | 1.28 (0.34, 4.79) | | | 1.28 (0.34, 4.79) | | | 1.46 (0.32, 6.73) | | | | 1.46 (0.32, 6.74) | | | | |
| BC (x10^-5^m^-1^) |  | | |  | | |  | | | |  | | | | |
| 0.8 vs 0.5 | 1.03 (0.56, 1.89) | | | 1.03 (0.56, 1.90) | | | 1.24 (0.60, 2.54) | | | | 1.24 (0.60, 2.55) | | | | |
| 1.2 vs 0.5 | 1.11 (0.50, 2.45) | | | 1.11 (0.50, 2.44) | | | 1.43 (0.56, 3.63) | | | | 1.45 (0.57, 3.68) | | | | |
| 1.6 vs 0.5 | 1.23 (0.37, 4.09) | | | 1.21 (0.36, 4.03) | | | 1.50 (0.40, 5.60) | | | | 1.54 (0.41, 5.74) | | | | |
| **Former smoker: quit > 10 years  before recruitment date** | | | **n = 5592** | |  | | | **n = 5591** | | | | | | | |
| PM_2.5_ (µg/m^3^) |  | | | | |  | | | |  | | | | | |
| 3 vs 1 | 1.86 (1.29, 2.69) | | | 1.87 (1.29, 2.72) | | | 1.13 (0.71, 1.80) | | | | 1.09 (0.68, 1.74) | | | | |
| 5 vs 1 | 2.40 (1.39, 4.15) | | | 2.39 (1.36, 4.18) | | | 1.12 (0.57, 2.21) | | | | 1.04 (0.52, 2.09) | | | | |
| 7 vs 1 | 1.52 (0.89, 2.59) | | | 1.43 (0.82, 2.52) | | | 0.87 (0.43, 1.76) | | | | 0.77 (0.36, 1.65) | | | | |
| NO_2_ (µg/m^3^) |  | | |  | | |  | | | |  | | | | |
| 10 vs 5 | 1.41 (1.00, 1.99) | | | 1.39 (0.99, 1.97) | | | 0.84 (0.53, 1.31) | | | | 0.83 (0.53, 1.30) | | | | |
| 15 vs 5 | 1.72 (1.04, 2.84) | | | 1.70 (1.02, 2.81) | | | 0.79 (0.41, 1.52) | | | | 0.78 (0.41, 1.51) | | | | |
| 25 vs 5 | 1.64 (0.93, 2.89) | | | 1.64 (0.94, 2.89) | | | 1.02 (0.46, 2.29) | | | | 1.03 (0.46, 2.29) | | | | |
| BC (x10^-5^m^-1^) |  | | |  | | | |  | | | | |  | | |
| 0.8 vs 0.5 | 1.44 (1.07, 1.92) | | | 1.42 (1.06, 1.90) | | | 0.92 (0.63, 1.35) | | | | 0.92 (0.63, 1.34) | | | | |
| 1.2 vs 0.5 | 1.49 (1.01, 2.18) | | | 1.46 (0.99, 2.15) | | | 1.02 (0.62, 1.69) | | | | 1.01 (0.61, 1.67) | | | | |
| 1.6 vs 0.5 | 1.09 (0.65, 1.85) | | | 1.07 (0.63, 1.82) | | | 1.32 (0.66, 2.67) | | | | 1.31 (0.65, 2.64) | | | | |
| **Former smoker: quit < 10 years  before recruitment date** | | | **n = 1315** | | |  | | | **n = 1328** | | | | | | |
| PM_2.5_ (µg/m^3^) |  | | | | |  | | | | | | |  | | |
| 3 vs 1 | 0.90 (0.61, 1.32) | | | 0.87 (0.58, 1.29) | | | 0.89 (0.39, 2.07) | | | | 1.04 (0.43, 2.49) | | | | |
| 5 vs 1 | 0.94 (0.50, 1.73) | | | 0.88 (0.46, 1.67) | | | 0.78 (0.21, 2.91) | | | | 1.04 (0.26, 4.12) | | | | |
| 7 vs 1 | 1.45 (0.80, 2.63) | | | 1.37 (0.73, 2.57) | | | 0.65 (0.17, 2.55) | | | | 0.92 (0.22, 3.86) | | | | |
| NO_2_ (µg/m^3^) |  | | |  | | | |  | | | | |  | | |
| 10 vs 5 | 0.97 (0.64, 1.47) | | | 0.97 (0.64, 1.47) | | | 1.71 (0.52, 5.61) | | | | 1.76 (0.53, 5.85) | | | | |
| 15 vs 5 | 0.95 (0.51, 1.77) | | | 0.95 (0.51, 1.77) | | | 2.43 (0.40, 14.80) | | | | 2.51 (0.41, 15.58) | | | | |
| 25 vs 5 | 0.92 (0.44, 1.92) | | | 0.93 (0.45, 1.94) | | | 2.54 (0.42, 15.25) | | | | 2.44 (0.41, 14.73) | | | | |
| BC (x10^-5^m^-1^) |  | | |  | | | | |  | | | | |  | |
| 0.8 vs 0.5 | 1.28 (0.86, 1.88) | | | 1.28 (0.87, 1.89) | | | 1.84 (0.65, 5.19) | | | | 1.83 (0.65, 5.18) | | | | |
| 1.2 vs 0.5 | 1.35 (0.79, 2.30) | | | 1.35 (0.79, 2.31) | | | 1.57 (0.39, 6.31) | | | | 1.56 (0.39, 6.27) | | | | |
| 1.6 vs 0.5 | 1.14 (0.58, 2.22) | | | 1.14 (0.58, 2.23) | | | 0.60 (0.09, 3.77) | | | | 0.59 (0.09, 3.74) | | | | |
|  |  | | |  | | | |  | | | | |  | | |
| **Current smoker** | **n = 1285** | | | | | | | **n = 1283** | | | | | | | |
| PM_2.5_ (µg/m^3^) |  | | | | |  | | | | | | |  | | |
| 3 vs 1 | 1.32 (0.88, 1.98) | | | 1.30 (0.86, 1.97) | | | 1.84 (0.60, 5.64) | | | | 2.00 (0.64, 6.29) | | | | |
| 5 vs 1 | 1.67 (0.84, 3.29) | | | 1.61 (0.80, 3.24) | | | 2.13 (0.34, 13.20) | | | | 2.50 (0.38, 16.30) | | | | |
| 7 vs 1 | 1.75 (0.91, 3.36) | | | 1.65 (0.83, 3.29) | | | 0.46 (0.07, 3.18) | | | | 0.58 (0.08, 4.35) | | | | |
| NO_2_ (µg/m^3^) |  | | |  | | | |  | | | | |  | | |
| 10 vs 5 | 0.88 (0.62, 1.25) | | | 0.88 (0.62, 1.24) | | | 0.75 (0.31, 1.79) | | | | 0.74 (0.31, 1.77) | | | | |
| 15 vs 5 | 0.86 (0.50, 1.48) | | | 0.86 (0.50, 1.48) | | | 0.74 (0.19, 2.82) | | | | 0.72 (0.19, 2.76) | | | | |
| 25 vs 5 | 1.30 (0.70, 2.43) | | | 1.34 (0.72, 2.51) | | | 2.08 (0.46, 9.43) | | | | 1.92 (0.42, 8.79) | | | | |
| BC (x10^-5^m^-1^) |  | | |  | | | | |  | | | | |  | |
| 0.8 vs 0.5 | 1.27 (0.88, 1.83) | | | 1.26 (0.87, 1.82) | | | 0.89 (0.40, 2.00) | | | | 0.90 (0.40, 2.02) | | | | |
| 1.2 vs 0.5 | 1.65 (0.98, 2.77) | | | 1.63 (0.97, 2.75) | | | 1.00 (0.32, 3.12) | | | | 1.01 (0.32, 3.15) | | | | |
| 1.6 vs 0.5 | 2.02 (1.13, 3.62) | | | 2.02 (1.13, 3.63) | | | 1.46 (0.33, 6.41) | | | | 1.43 (0.33, 6.27) | | | | |

*^a^Hazard ratios considered reference values of: 1µg/m^3^ for PM_2.5_, 5µg/m^3^for NO_2_ , and 0.5x10^-5^m^-1^ for BC. Age used as the timescale.*

### Table S4. Sensitivity analysis using pollutant concentrations assigned to participants at recruitment (baseline), lung cancer and bladder cancer analysis in the HIMS cohort

Associations between incident lung and bladder cancer and ambient air pollution are described below, where the exposure metric is the air pollutant concentrations assigned to participants in the year of recruitment (at baseline). Baseline exposure was estimated by calculating the average concentration of each pollutant during the participant’s recruitment year. Single-pollutant adjusted hazard ratios were estimated for PM_2.5_, NO_2_, and BC. Age was used as the timescale.

| **Air pollutant**  **concentration^a^** | **Total lung cancer cohort  (n=692, total person-years = 158,479)** | | | | **Total bladder cancer cohort  (n=224, total person-years = 158,440)** | | | | |
| --- | --- | --- | --- | --- | --- | --- | --- | --- | --- |
|  | **Unadjusted** | **Adjusted^b^** | | **Unadjusted** | | | **Adjusted^b^** | |  |
|  | **HR (95%CI)** | **HR (95%CI)** | | **HR (95%CI)** | | | **HR (95%CI)** | |  |
| PM_2.5_ (µg/m^3^) |  |  | |  | | |  | |  |
| 3 vs 1 | 1.35 (1.10, 1.65) | 1.24 (1.01, 1.53) | | 1.15 (0.83, 1.59) | | | 1.14 (0.82, 1.59) | |  |
| 5 vs 1 | 1.74 (1.23, 2.46) | 1.47 (1.03, 2.08) | | 1.26 (0.73, 2.17) | | | 1.24 (0.71, 2.16) | |  |
| 7 vs 1 | 1.91 (1.38, 2.65) | 1.44 (1.03, 2.02) | | 1.14 (0.68, 1.91) | | | 1.13 (0.66, 1.93) | |  |
| NO_2_ (µg/m^3^) |  |  | |  | | |  | |  |
| 10 vs 5 | 1.20 (0.97, 1.50) | 1.10 (0.89, 1.37) | | 0.93 (0.65, 1.33) | | | 0.91 (0.63, 1.30) | |  |
| 15 vs 5 | 1.35 (0.97, 1.86) | 1.17 (0.85, 1.61) | | 0.95 (0.56, 1.62) | | | 0.93 (0.54, 1.57) | |  |
| 25 vs 5 | 1.33 (0.92, 1.94) | 1.17 (0.80, 1.70) | | 1.46 (0.79, 2.68) | | | 1.41 (0.77, 2.59) | |  |
| BC (x10^-5^m^-1^) |  | |  | | |  | |  |  |
| 0.8 vs 0.5 | 1.35 (1.11, 1.64) | 1.25 (1.03, 1.51) | | 1.05 (0.77, 1.43) | | | 1.03 (0.76, 1.41) | |  |
| 1.2 vs 0.5 | 1.54 (1.19, 1.98) | 1.35 (1.04, 1.74) | | 1.16 (0.77, 1.75) | | | 1.14 (0.75, 1.72) | |  |
| 1.6 vs 0.5 | 1.41 (1.01, 1.97) | 1.22 (0.88, 1.71) | | 1.33 (0.76, 2.32) | | | 1.30 (0.75, 2.28) | |  |

*^a^Hazard ratios considered reference values of: 1µg/m^3^ for PM_2.5_, 5µg/m^3^ for NO_2_ , and 0.5x10^-5^m^-1^ for BC
 ^b^Adjusted for smoking status and socioeconomic status*

### Table S5. Sensitivity analysis using pollutant concentrations assigned to participants at recruitment (baseline), lung histological subtype analysis in the HIMS cohort

Associations between incident squamous cell lung carcinoma and lung adenocarcinoma, and ambient air pollution are described below. Air pollutant exposures in the year of recruitment (at baseline) were assigned to participants. This was estimated by calculating the average concentration of each pollutant during the participant’s recruitment year. Single-pollutant adjusted hazard ratios were estimated for PM_2.5_, NO_2_, and BC. Age was used as the timescale.

| **Air pollutant**  **concentration^a^** | **Squamous cell lung carcinoma  (n=138, total person-years = 159,190)** | | | | **Lung adenocarcinoma  (n=201, total person- years = 159,113)** | | | | |
| --- | --- | --- | --- | --- | --- | --- | --- | --- | --- |
|  | **Unadjusted** | **Adjusted^b^** | | **Unadjusted** | | | **Adjusted^b^** | |  |
|  | **HR (95%CI)** | **HR (95%CI)** | | **HR (95%CI)** | | | **HR (95%CI)** | |  |
| PM_2.5_ (µg/m^3^) |  |  | |  | | |  | |  |
| 3 vs 1 | 1.38 (0.86, 2.20) | 1.23 (0.77, 1.97) | | 1.17 (0.83, 1.66) | | | 1.10 (0.78, 1.56) | |  |
| 5 vs 1 | 1.84 (0.83, 4.10) | 1.46 (0.65, 3.27) | | 1.29 (0.72, 2.31) | | | 1.12 (0.62, 2.02) | |  |
| 7 vs 1 | 2.26 (1.07, 4.79) | 1.52 (0.70, 3.33) | | 1.15 (0.67, 1.99) | | | 0.87 (0.49, 1.55) | |  |
| NO_2_ (µg/m^3^) |  |  | |  | | |  | |  |
| 10 vs 5 | 1.14 (0.70, 1.86) | 1.02 (0.63, 1.65) | | 1.01 (0.69, 1.48) | | | 0.95 (0.65, 1.38) | |  |
| 15 vs 5 | 1.31 (0.63, 2.70) | 1.10 (0.54, 2.23) | | 0.98 (0.56, 1.72) | | | 0.88 (0.51, 1.53) | |  |
| 25 vs 5 | 1.78 (0.80, 3.98) | 1.51 (0.68, 3.37) | | 0.81 (0.40, 1.65) | | | 0.72 (0.35, 1.48) | |  |
| BC (x10^-5^m^-1^) |  | |  | | |  | |  |  |
| 0.8 vs 0.5 | 1.47 (0.93, 2.33) | 1.33 (0.84, 2.11) | | 1.23 (0.87, 1.74) | | | 1.16 (0.82, 1.64) | |  |
| 1.2 vs 0.5 | 1.94 (1.06, 3.57) | 1.64 (0.88, 3.03) | | 1.34 (0.85, 2.12) | | | 1.20 (0.76, 1.90) | |  |
| 1.6 vs 0.5 | 2.11 (1.01, 4.40) | 1.75 (0.84, 3.65) | | 1.25 (0.68, 2.32) | | | 1.10 (0.59, 2.04) | |  |

*^a^Hazard ratios considered reference values of: 1µg/m^3^ for PM_2.5_, 5µg/m^3^ for NO_2_ , and 0.5x10^-5^m^-1^ for BC
^b^Adjusted for smoking status and socioeconomic status*

### Table S6. Sensitivity analysis excluding lung and bladder cancer incidence during the first two years of follow-up in the HIMS cohort

The table below shows the associations between ambient air pollutant concentrations, and incident lung and bladder cancer. Participants who were diagnosed with lung or bladder cancer in the first two years of follow-up were excluded in the respective analyses. Single pollutant unadjusted and adjusted hazard ratios were estimated.

| **Air pollutant**  **concentration^a^** | **Total lung cancer cohort  (N = 11 541)** | | | | **Total bladder cancer cohort  (N = 11 603)** | | | | |
| --- | --- | --- | --- | --- | --- | --- | --- | --- | --- |
|  | **Unadjusted** | **Adjusted^b^** | | **Unadjusted** | | | **Adjusted^b^** | |  |
|  | **HR (95%CI)** | **HR (95%CI)** | | **HR (95%CI)** | | | **HR (95%CI)** | |  |
| PM_2.5_ (µg/m^3^) |  |  | |  | | |  | |  |
| 3 vs 1 | 1.52 (1.19, 1.94) | 1.38 (1.08, 1.76) | | 1.34 (0.90, 1.99) | | | 1.32 (0.88, 1.97) | |  |
| 5 vs 1 | 1.99 (1.38, 2.85) | 1.64 (1.13, 2.37) | | 1.46 (0.82, 2.59) | | | 1.42 (0.78, 2.56) | |  |
| 7 vs 1 | 1.98 (1.40, 2.80) | 1.47 (1.02, 2.12) | | 1.08 (0.60, 1.94) | | | 1.03 (0.55, 1.92) | |  |
| NO_2_ (µg/m^3^) |  |  | |  | | |  | |  |
| 10 vs 5 | 1.23 (0.98, 1.54) | 1.15 (0.92, 1.44) | | 1.00 (0.69, 1.44) | | | 0.98 (0.68, 1.42) | |  |
| 15 vs 5 | 1.41 (1.01, 1.95) | 1.27 (0.92, 1.76) | | 1.05 (0.61, 1.79) | | | 1.02 (0.60, 1.74) | |  |
| 25 vs 5 | 1.50 (1.03, 2.18) | 1.38 (0.95, 2.01) | | 1.38 (0.73, 2.61) | | | 1.33 (0.70, 2.51) | |  |
| BC (x10^-5^m^-1^) |  | |  | | |  | |  |  |
| 0.8 vs 0.5 | 1.39 (1.14, 1.70) | 1.31 (1.07, 1.60) | | 1.16 (0.84, 1.59) | | | 1.14 (0.82 (1.57) | |  |
| 1.2 vs 0.5 | 1.60 (1.23, 2.07) | 1.46 (1.12, 1.90) | | 1.28 (0.84, 1.96) | | | 1.25 (0.82, 1.92) | |  |
| 1.6 vs 0.5 | 1.45 (1.03, 2.04) | 1.35 (0.96, 1.90) | | 1.34 (0.74, 2.41) | | | 1.30 (0.72, 2.34) | |  |

*^a^Hazard ratios considered reference values of: 1µg/m^3^ for PM_2.5_, 5µg/m^3^ for NO_2_ , and 0.5x10^-5^m^-1^ for BC
^b^Adjusted for smoking status and socioeconomic status*

### Table S7. Sensitivity analysis excluding movers in the HIMS cohort

Associations between ambient air pollutant concentrations, and incident lung and bladder cancer are described below. Participants who moved residential addresses during recruitment were excluded in the respective analyses. There were 1139 and 1140 participants that moved in the lung and bladder cancer cohorts respectively, and therefore, were excluded.

| **Air pollutant**  **concentration^a^** | **Lung cancer (N = 10 478)** | | | **Bladder cancer (N = 10 487)** | | | | |
| --- | --- | --- | --- | --- | --- | --- | --- | --- |
|  | **Unadjusted** | **Adjusted^b^** | | | **Unadjusted** | | **Adjusted^b^** | |
|  | **HR (95% CI)** | **HR (95% CI)** | | | **HR (95% CI)** | | **HR (95% CI)** | |
| PM_2.5_ (µg/m^3^) |  | |  | | |  | | |
| 3 vs 1 | 1.34 (1.06, 1.68) | 1.26 (1.00, 1.59) | | | 1.05 (0.73, 1.50) | | 1.05 (0.73, 1.52) | |
| 5 vs 1 | 1.67 (1.18, 2.36) | 1.45 (1.01, 2.06) | | | 1.03 (0.61, 1.75) | | 1.03 (0.59, 1.78) | |
| 7 vs 1 | 1.81 (1.30, 2.52) | 1.37 (0.96, 1.95) | | | 0.90 (0.52, 1.56) | | 0.88 (0.49, 1.58) | |
| NO_2_ (µg/m^3^) |  |  | | |  | |  | |
| 10 vs 5 | 1.16 (0.93, 1.44) | 1.09 (0.88, 1.36) | | | 0.95 (0.66, 1.37) | | 0.93 (0.65, 1.35) | |
| 15 vs 5 | 1.28 (0.92 ,1.77) | 1.17 (0.85, 1.62) | | | 1.00 (0.58, 1.71) | | 0.97 (0.57, 1.67) | |
| 25 vs 5 | 1.37 (0.95, 1.98) | 1.28 (0.88, 1.84) | | | 1.56 (0.85, 2.89) | | 1.50 (0.81, 2.77) | |
| BC (x10^-5^m^-1^) |  | |  | | |  | |  |
| 0.8 vs 0.5 | 1.37 (1.12, 1.67) | 1.29 (1.06, 1.58) | | | 1.02 (0.75, 1.39) | | 1.01 (0.74, 1.38) | |
| 1.2 vs 0.5 | 1.59 (1.22, 2.08) | 1.46 (1.12, 1.92) | | | 1.12 (0.74, 1.69) | | 1.09 (0.72, 1.65) | |
| 1.6 vs 0.5 | 1.52 (1.09, 2.11) | 1.41 (1.01, 1.96) | | | 1.29 (0.73, 2.26) | | 1.25 (0.71, 2.19) | |

*^a^Hazard ratios considered reference values of: 1µg/m^3^ for PM_2.5_, 5µg/m^3^ for NO_2_ , and 0.5x10^-5^m^-1^ for BC. Age was used as the timescale
^b^Adjusted for smoking status and socioeconomic status*

### Table S8. Sensitivity analysis adjusted for all available covariates in the HIMS cohort

Associations between ambient air pollutants and incident lung and bladder cancer are described below. Single-pollutant adjusted models have included all available covariates: smoking status, job status, education status and physical activity. Participants with missing information on the additional covariates were excluded. The unadjusted models were also re-run, to enable comparison of hazard ratios with the adjusted models. Minimal changes in our adjusted estimates are expected, as the number of the additional participants removed from these analyses were few (n=7).

| **Air pollutant**  **concentration^a^** | **Lung cancer (n=11 610)** | | | **Bladder cancer (n= 11 620)** | | |
| --- | --- | --- | --- | --- | --- | --- |
|  | **Unadjusted** | **Adjusted** | | **Unadjusted** | | **Adjusted** |
|  | **HR (95% CI)** | **HR (95% CI)** | | **HR (95% CI)** | | **HR (95% CI)** |
| PM_2.5_ (µg/m^3^) |  | |  | |  | |
| 3 vs 1 | 1.38 (1.10, 1.71) | 1.21 (0.96, 1.52) | | 1.20 (0.84, 1.70) | | 1.16 (0.81, 1.68) |
| 5 vs 1 | 1.76 (1.26, 2.46) | 1.36 (0.96, 1.93) | | 1.27 (0.75, 2.14) | | 1.21 (0.69, 2.11) |
| 7 vs 1 | 1.95 (1.42, 2.69) | 1.34 (0.94, 1.89) | | 1.05 (0.62, 1.79) | | 0.99 (0.55, 1.78) |
| NO_2_ (µg/m^3^) |  |  | |  | |  |
| 10 vs 5 | 1.15 (0.93, 1.42) | 1.06 (0.86, 1.31) | | 0.99 (0.70, 1.41) | | 0.97 (0.68, 1.38) |
| 15 vs 5 | 1.28 (0.94, 1.75) | 1.13 (0.83, 1.54) | | 1.04 (0.62, 1.74) | | 0.99 (0.59, 1.67) |
| 25 vs 5 | 1.45 (1.02, 2.05) | 1.29 (0.91, 1.84) | | 1.38 (0.76, 2.51) | | 1.28 (0.70, 2.33) |
| BC (x10^-5^m^-1^)  0.8 vs 0.5 | 1.38 (1.15, 1.67) | 1.28 (1.05, 1.55) | | 1.08 (0.80, 1.45) | | 1.06 (0.78, 1.43) |
| 1.2 vs 0.5 | 1.59 (1.24, 2.04) | 1.40 (1.09, 1.81) | | 1.18 (0.79, 1.75) | | 1.13 (0.75, 1.69) |
| 1.6 vs 0.5 | 1.47 (1.07, 2.02) | 1.30 (0.94, 1.80) | | 1.28 (0.74, 2.20) | | 1.21 (0.70, 2.10) |

*^a^Hazard ratios considered reference values of: 1µg/m^3^ for PM_2.5_, 5µg/m^3^for NO_2_ , and 0.5x10^-5^m^-1^ for BC. Age used as the timescale.*

### Figure S1. Directed acyclic graph describing association between confounding factors and lung cancer

Directed Acyclic Graphs (DAG) were developed to identify the minimum adjustment set required for confounding between ambient air pollution and lung cancer. From the DAG below, the minimal sufficient adjustment sets for estimating the total effect of ambient air pollution on lung cancer include age and socioeconomic status.

*
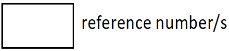
***
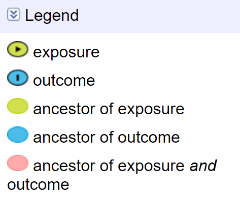
**
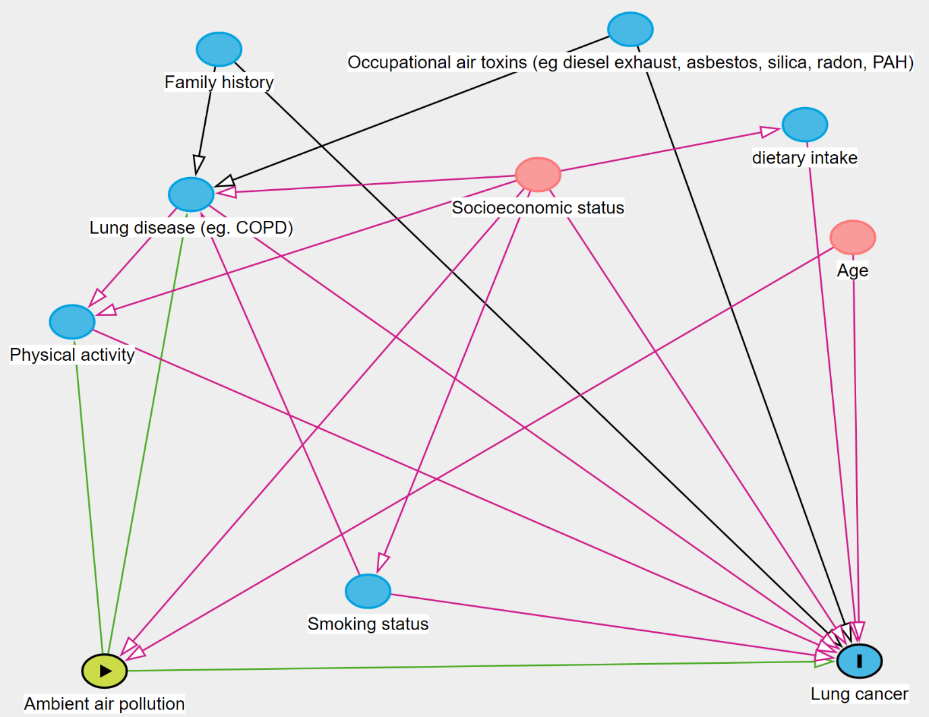


12,13

14,15

16-18

3-5

6-9

41

34,35

30,31

24

43-45

19

42

20,21

22,23

27-29

25,26

10,11

38-40

36,37

32,33

46

### Figure S2. Directed acyclic graph describing association between confounding factors and bladder cancer

Directed Acyclic Graphs (DAG) were developed to identify the minimum adjustment set required for confounding between ambient air pollution and bladder cancer. From the DAG below, the minimal sufficient adjustment sets for estimating the total effect of ambient air pollution on bladder cancer include age, smoking statuss and socioeconomic status.

*
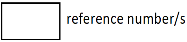
***
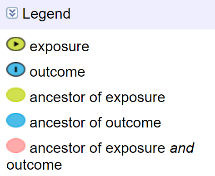
**
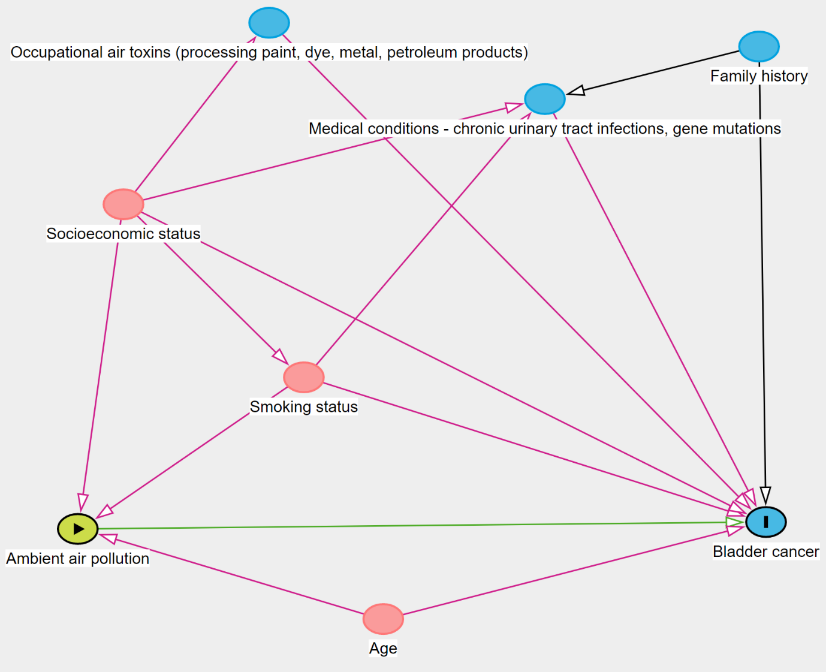


4042

2830

43,6245,64

53,5455,56

28,2930,31

4749

5961

48-50-52

51,5253,54

55,5657,58

57,5859,60

45,4647,48

2224

60,6162,63

6365

### Figure S3. Flowchart of the HIMS recruitment and selection process

The flowchart describes the numbers of participants recruited to the HIMS Study, and the total number of participants eligible for the lung cancer and bladder analysis.

Participants eligible for current study (n=11 679)

Participants enrolled in HIMS Study at Wave 1: 1996-1999
(n=12 203)

Excluded (n=524)

♦  Lived outside metropolitan Perth (n=476)

♦  Missing data on key baseline variables:

- Smoking status (n=43)

- Socioeconomic status (n=5)

Separate into 2 Analysis

Bladder cancer cohort (n=11 627)

♦ Prior bladder cancer excluded (n=52)

Lung cancer cohort (n=11 617)

♦ Prior lung cancer excluded (n=62)

Analyse association between bladder cancer and PM_2.5_, NO_2_ and BC

Analyse association between lung cancer and PM_2.5_, NO_2_ and BC

Histological subgroup analysis (n=11 617)
♦ Squamous cell carcinoma
♦ Adenocarcinoma

### Figure S4. Graphs of 3-knot restricted cubic spline regression using air pollutant concentrations of HIMS participants at recruitment

The spline graphs below demonstrate the non-linear associations between ambient air pollutants and incident lung cancer, bladder cancer, and histological subtypes of lung cancer (adenocarcinoma “ADC” and squamous cell carcinoma “SCC”). The exposure metrics assigned to participants is the average concentrations in their year of recruitment into the HIMS Study.

| i) Lung cancer: PM_2.5_ | ii) Lung cancer: NO_2_ | iii) Lung cancer: BC |
| --- | --- | --- |
| 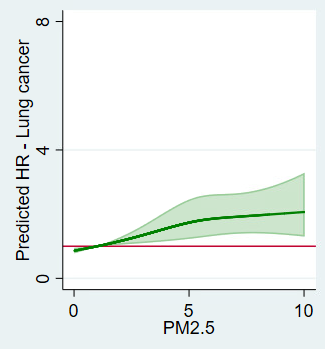 | 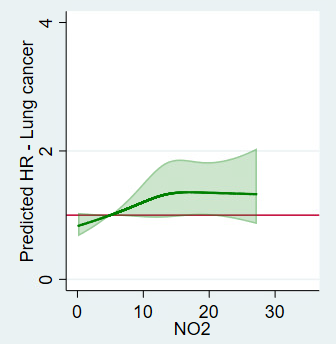 | 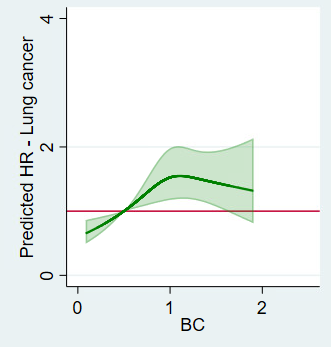 |
| iv) Bladder cancer: PM_2.5_ | v) Bladder cancer: NO_2_ | vi) Bladder cancer: BC |
| 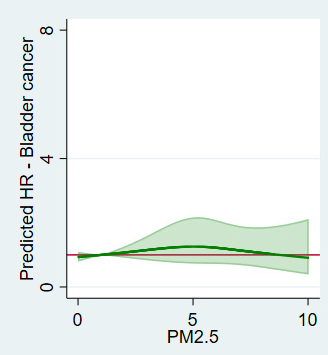 | 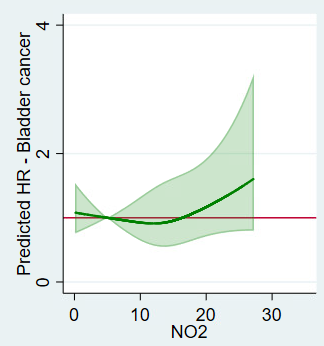 | 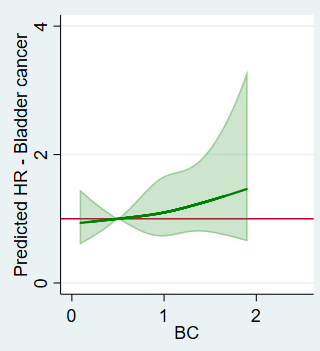 |
| vii) ADC: PM_2.5_ | viii) ADC: NO_2_ | iv) ADC: BC |
| 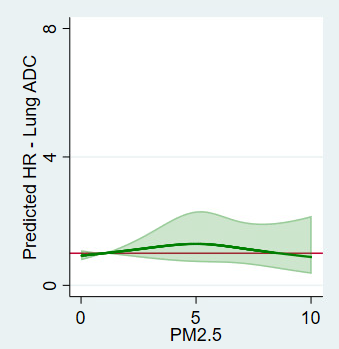 | 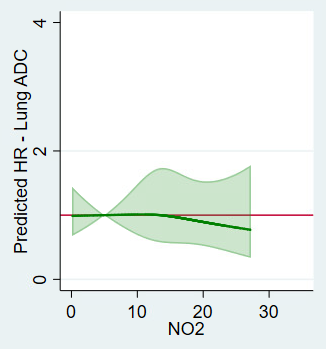 | 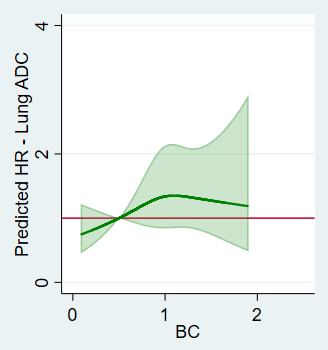 |
| x) SCC: PM_2.5_ | xi) SCC: NO_2_ | xii) SCC: BC |
| 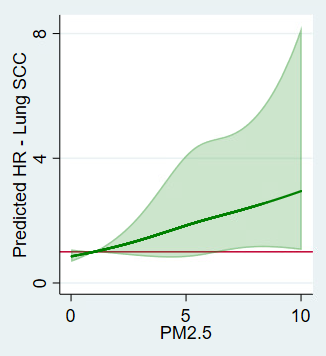 | 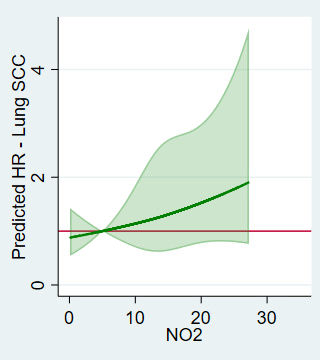 | 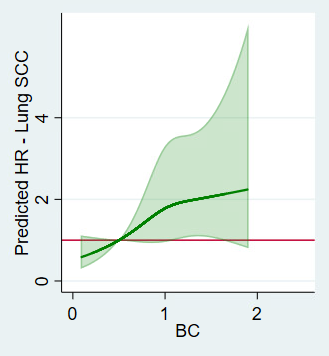 |

### References

1. Strijk J. Health (Western Australian Cancer Register) Regulations 2011. In: Gazette WAG, editor. 72. Perth (WA): State of Western Australia; 2011. p. 17.

2. Data Quality Statement - Western Australian Cancer Register (Government of Western Australia) 11 (2020).

3. An R, Shen J, Ying B, et al. Impact of ambient air pollution on physical activity and sedentary behavior in China: A systematic review. Environ Res [Internet]. 2019;176:108545. Available from: <https://pubmed.ncbi.nlm.nih.gov/31280030/>. doi: 10.1016/j.envres.2019.108545

4. An R, Zhang S, Ji M, et al. Impact of ambient air pollution on physical activity among adults: a systematic review and meta-analysis. Perspect Public Health [Internet]. 2018;138(2):111-121. Available from: <https://journals.sagepub.com/doi/10.1177/1757913917726567>. doi: 10.1177/1757913917726567

5. Tainio M, Jovanovic Andersen Z, Nieuwenhuijsen MJ, et al. Air pollution, physical activity and health: A mapping review of the evidence. Environ Int [Internet]. 2021;147:105954. Available from: <https://www.sciencedirect.com/science/article/pii/S0160412020319097?via%3Dihub>. doi: 10.1016/j.envint.2020.105954

6. Doiron D, Bourbeau J, de Hoogh K, et al. Ambient air pollution exposure and chronic bronchitis in the Lifelines cohort. Thorax [Internet]. 2021;76(8):772. Available from: <https://thorax.bmj.com/content/76/8/772>. doi:10.1136/thoraxjnl-2020-216142.

7. Wang M, Aaron CP, Madrigano J, et al. Association Between Long-term Exposure to Ambient Air Pollution and Change in Quantitatively Assessed Emphysema and Lung Function. JAMA [Internet]. 2019;322(6):546-556. Available from: <https://pubmed.ncbi.nlm.nih.gov/31408135/>. doi: 10.1001/jama.2019.10255

8. Holst GJ, Pedersen CB, Thygesen M, et al. Air pollution and family related determinants of asthma onset and persistent wheezing in children: nationwide case-control study. BMJ [Internet]. 2020;370:m2791. Available from: <https://www.bmj.com/content/370/bmj.m2791>. doi: 10.1136/bmj.m2791

9. Thurston GD, Balmes JR, Garcia E, et al. Outdoor Air Pollution and New-Onset Airway Disease. An Official American Thoracic Society Workshop Report. Ann Am Thorac Soc [Internet]. 2020;17(4):387-398. Available from: https://www.atsjournals.org/doi/10.1513/AnnalsATS.202001-046ST. doi: 10.1513/AnnalsATS.202001-046ST

10. Hersh CP, Hokanson JE, Lynch DA, et al. Family history is a risk factor for COPD. Chest [Internet]. 2011;140(2):343-350. Available from: <https://pubmed.ncbi.nlm.nih.gov/21310839/>. doi: 10.1378/chest.10-2761

11. Yu H, Su F, Wang L-B, et al. The Asthma Family Tree: Evaluating Associations Between Childhood, Parental, and Grandparental Asthma in Seven Chinese Cities. Front Pediatr [Internet]. 2021;9. Available from: <https://www.frontiersin.org/articles/10.3389/fped.2021.720273/full>. doi: 10.3389/fped.2021.720273

12. Kanwal M, Ding X-J, Cao Y. Familial risk for lung cancer (Review). Oncol Lett [Internet]. 2017;13(2):535-542. Available from: <https://www.spandidos-publications.com/10.3892/ol.2016.5518>. doi: 10.3892/ol.2016.5518

13. Matakidou A, Eisen T, Houlston RS. Systematic review of the relationship between family history and lung cancer risk. Br J Cancer [Internet]. 2005;93(7):825-833. Available from: <https://pubmed.ncbi.nlm.nih.gov/16160696/>. doi: 10.1038/sj.bjc.6602769

14. Cullinan P. Occupation and chronic obstructive pulmonary disease (COPD). Br Med Bull [Internet]. 2012;104(1):143-161. Available from: <https://pubmed.ncbi.nlm.nih.gov/23080418/>. doi: 10.1093/bmb/lds028

15. Connellan SJ. Lung diseases associated with hydrocarbon exposure. Respir Med [Internet]. 2017;126:46-51. Available from: <https://www.sciencedirect.com/science/article/pii/S0954611117300926?via%3Dihub>. doi: 10.1016/j.rmed.2017.03.021

16. Shankar A, Dubey A, Saini D, et al. Environmental and occupational determinants of lung cancer. Transl Lung Cancer Res [Internet]. 2019;8(Suppl 1):S31-S49. Available from: <https://pubmed.ncbi.nlm.nih.gov/31211104/>. doi: 10.21037/tlcr.2019.03.05

17. Carlsten C, Georas SN. Update in Environmental and Occupational Lung Diseases 2013. Am J Respir Crit Care Med [Internet]. 2014;189(9):1037-1043. Available from: <https://www.atsjournals.org/doi/full/10.1164/rccm.201401-0108UP>. doi: 10.1164/rccm.201401-0108UP

18. Warden H, Richardson H, Richardson L, et al. Associations between occupational exposure to benzene, toluene and xylene and risk of lung cancer in Montréal. Occup Environ Med [Internet]. 2018;75(10):696-702. Available from: <https://oem.bmj.com/content/75/10/696>. doi: 10.1136/oemed-2017-104987

19. Sun Y, Li Z, Li J, et al. A Healthy Dietary Pattern Reduces Lung Cancer Risk: A Systematic Review and Meta-Analysis. Nutrients [Internet]. 2016;8(3):134. Available from: <https://pubmed.ncbi.nlm.nih.gov/26959051/>. doi: 10.3390/nu8030134

20. Sahni S, Talwar A, Khanijo S, et al. Socioeconomic status and its relationship to chronic respiratory disease. Adv Respir Med [Internet]. 2017;85(2):97-108. Available from: https://pubmed.ncbi.nlm.nih.gov/28440535/. doi: 10.5603/arm.2017.0016

21. Kanervisto M, Vasankari T, Laitinen T, et al. Low socioeconomic status is associated with chronic obstructive airway diseases. Respir Med [Internet]. 2011;105(8):1140-1146. Available from: <https://www.sciencedirect.com/science/article/pii/S0954611111000965?via%3Dihub>. doi: https://doi.org/10.1016/j.rmed.2011.03.008

22. Stalsberg R, Pedersen AV. Are Differences in Physical Activity across Socioeconomic Groups Associated with Choice of Physical Activity Variables to Report? Int J Environ Res Public Health [Internet]. 2018;15(5):922. Available from: <https://pubmed.ncbi.nlm.nih.gov/29734745/>. doi: 10.3390/ijerph15050922

23. Christie CD, Consoli A, Ronksley PE, et al. Associations between the built environment and physical activity among adults with low socio-economic status in Canada: a systematic review. Can J Public Health [Internet]. 2021;112(1):152-165. Available from: <https://link.springer.com/article/10.17269/s41997-020-00364-9>. doi: 10.17269/s41997-020-00364-9

24. Hitchman SC, Fong GT, Zanna MP, et al. Socioeconomic status and smokers' number of smoking friends: findings from the International Tobacco Control (ITC) Four Country Survey. Drug Alcohol Depend [Internet]. 2014;143:158-166. Available from: <https://pubmed.ncbi.nlm.nih.gov/25156228/>. doi: 10.1016/j.drugalcdep.2014.07.019

25. Hovanec J, Siemiatycki J, Conway DI, et al. Lung cancer and socioeconomic status in a pooled analysis of case-control studies. PLoS One. 2018;13(2):e0192999. Available from: <https://journals.plos.org/plosone/article?id=10.1371/journal.pone.0192999>. doi: 10.1371/journal.pone.0192999

26. Ekberg-Aronsson M, Nilsson PM, Nilsson JA, et al. Socio-economic status and lung cancer risk including histologic subtyping -a longitudinal study. Lung Cancer [Internet]. 2006;51(1):21-29. Available from: <https://pubmed.ncbi.nlm.nih.gov/16337709/>. doi: 10.1016/j.lungcan.2005.08.014

27. Pechey R, Monsivais P. Socioeconomic inequalities in the healthiness of food choices: Exploring the contributions of food expenditures. Prev Med [Internet]. 2016;88:203-209. Available from: <https://pubmed.ncbi.nlm.nih.gov/27095324/>. doi: 10.1016/j.ypmed.2016.04.012

28. Hulshof KFAM, Brussaard JH, Kruizinga AG, et al. Socio-economic status, dietary intake and 10 y trends: the Dutch National Food Consumption Survey. Eur J Clin Nutr [Internet]. 2003;57(1):128-137. Available from: <https://pubmed.ncbi.nlm.nih.gov/12548307/>. doi: 10.1038/sj.ejcn.1601503

29. Vlismas K, Stavrinos V, Panagiotakos DB. Socio-economic status, dietary habits and health-related outcomes in various parts of the world: a review. Cent Eur J Public Health [Internet]. 2009;17(2):55-63. Available from: <https://europepmc.org/article/MED/19662821>. doi: 10.21101/cejph.a3475

30. Hajat A, Hsia C, O’Neill MS. Socioeconomic disparities and air pollution exposure: a global review. Curr Environ Health Rep [Internet]. 2015;2(4):440-450. Available from: <https://www.ncbi.nlm.nih.gov/pmc/articles/PMC4626327/>. doi: 10.1007/s40572-015-0069-5

31. Jiao K, Xu M, Liu M. Health status and air pollution related socioeconomic concerns in urban China. Int J Equity Health [Internet]. 2018;17(1):18. Available from: <https://equityhealthj.biomedcentral.com/articles/10.1186/s12939-018-0719-y>. doi: 10.1186/s12939-018-0719-y

32. Shin K-C. Physical activity in chronic obstructive pulmonary disease: clinical impact and risk factors. Korean J Intern Med [Internet]. 2018;33(1):75-77. Available from: <https://pubmed.ncbi.nlm.nih.gov/29334725/>. doi: 10.3904/kjim.2017.387

33. Strine TW, Balluz LS, Ford ES. The Associations between Smoking, Physical Inactivity, Obesity, and Asthma Severity in the General US Population. J Asthma [Internet]. 2007;44(8):651-658. Available from: <https://www.tandfonline.com/doi/abs/10.1080/02770900701554896>. doi: 10.1080/02770900701554896

34. Balbi B, Cottin V, Singh S, et al. Smoking-related lung diseases: a clinical perspective. Eur Respir J [Internet]. 2010;35(2):231. Available from: <https://pubmed.ncbi.nlm.nih.gov/20123840/>. doi: 10.1183/09031936.00189309

35. Sousa C, Rodrigues M, Carvalho A, et al. Diffuse smoking-related lung diseases: insights from a radiologic-pathologic correlation. Insights Imaging [Internet]. 2019;10(1):73. Available from: <https://www.ncbi.nlm.nih.gov/pmc/articles/PMC6635572/>. doi: 10.1186/s13244-019-0765-z

36. Denholm R, Schüz J, Straif K, et al. Is previous respiratory disease a risk factor for lung cancer? Am J Respir Crit Care Med [Internet]. 2014;190(5):549-559. Available from: <https://www.atsjournals.org/doi/full/10.1164/rccm.201402-0338OC>. doi: 10.1164/rccm.201402-0338OC

37. Husebø GR, Nielsen R, Hardie J, et al. Risk factors for lung cancer in COPD – results from the Bergen COPD cohort study. Respir Med [Internet]. 2019;152:81-88. Available from: <https://pubmed.ncbi.nlm.nih.gov/29632898/>. doi.org/10.1016/j.rmed.2019.04.019

38. Cannioto R, Etter JL, LaMonte MJ, et al. Lifetime physical inactivity is associated with lung cancer risk and mortality. Cancer Treat Res Commun [Internet]. 2018;14:37-45. Available from: doi: 10.1016/j.ctarc.2018.01.001

39. Jiang L, Sun Y-Q, Brumpton BM, et al. Prolonged Sitting, Its Combination With Physical Inactivity and Incidence of Lung Cancer: Prospective Data From the HUNT Study. Front Oncol [Internet]. 2019;9:101. Available from: <https://pubmed.ncbi.nlm.nih.gov/30859092/>. doi: 10.3389/fonc.2019.00101

40. Zhong S, Ma T, Chen L, et al. Physical Activity and Risk of Lung Cancer: A Meta-analysis. Clin J Sport Med [Internet]. 2016;26(3):173-181. Available from: https://pubmed.ncbi.nlm.nih.gov/26213983/. doi: 10.1097/JSM.0000000000000219

41. Doll R, Hill AB. Smoking and carcinoma of the lung; preliminary report. Br Med J [Internet]. 1950;2(4682):739-748. Available from: <https://www.ncbi.nlm.nih.gov/pmc/articles/PMC2038856/>. doi: 10.1136/bmj.2.4682.739

42. Ward-Caviness CK, Nwanaji-Enwerem JC, Wolf K, et al. Long-term exposure to air pollution is associated with biological aging. Oncotarget [Internet]. 2016;7(46):74510-74525. Available from: <https://pubmed.ncbi.nlm.nih.gov/27793020/>. doi: 10.18632/oncotarget.12903

43. O'Rourke MA, Feussner JR, Feigl P, et al. Age trends of lung cancer stage at diagnosis. Implications for lung cancer screening in the elderly. JAMA [Internet]. 1987;258(7):921-926. Available from: <https://pubmed.ncbi.nlm.nih.gov/3613022/>

44. Cancer Australia. Lung Cancer in Australia statistics [Internet]. [Strawberry Hills (NSW)]: Commonwealth of Australia; c2021. [revised 2020 Oct 20; cited 2021 Mar 9]. Available from: <https://www.canceraustralia.gov.au/affected-cancer/cancer-types/lung-cancer/lung-cancer-australia-statistics>

45. DeSantis CE, Miller KD, Dale W, et al. Cancer statistics for adults aged 85 years and older, 2019. CA: Cancer J Clin [Internet]. 2019;69(6):452-467. Available from: <https://acsjournals.onlinelibrary.wiley.com/doi/full/10.3322/caac.21577>. doi: 10.3322/caac.21577

46. World Health Organization. WHO global air quality guidelines: particulate matter (‎PM2.5 and PM10)‎, ozone, nitrogen dioxide, sulfur dioxide and carbon monoxide [Internet]. Geneva: World Health Organization; 2021 [cited 2021 Oct 15]. 273 p. Available from: <https://apps.who.int/iris/handle/10665/345329>

47. Densmore R, Hajizadeh M, Hu M. Trends in socio-economic inequalities in bladder cancer incidence in Canada: 1992–2010. Can J Public Health [Internet]. 2019;110(6):722-731. Available from: <https://www.ncbi.nlm.nih.gov/pmc/articles/PMC6964608/>. doi: 10.17269/s41997-019-00227-y

48. Mihor A, Tomsic S, Zagar T, et al. Socioeconomic inequalities in cancer incidence in Europe: a comprehensive review of population-based epidemiological studies. Radiol Oncol [Internet]. 2020;54(1):1-13. Available from: <https://pubmed.ncbi.nlm.nih.gov/32074075/>. doi: 10.2478/raon-2020-0008

49. Casey JA, Rudolph KE, Robinson SC, et al. Sociodemographic Inequalities in Urinary Tract Infection in 2 Large California Health Systems. Open Forum Infect Dis [Internet]. 2021;8(6). Available from: https://pubmed.ncbi.nlm.nih.gov/34189179/. doi: 10.1093/ofid/ofab276

50. Reed O, Jubber I, Griffin J, et al. Occupational bladder cancer: A cross section survey of previous employments, tasks and exposures matched to cancer phenotypes. PLoS One. 2020;15(10):e0239338. Available from: https://journals.plos.org/plosone/article?id=10.1371/journal.pone.0239338. doi: 10.1371/journal.pone.0239338

51. Clavel J, Mandereau L, Limasset J, et al. Occupational Exposure to Polycyclic Aromatic Hydrocarbons and the Risk of Bladder Cancer: A French Case-Control Study. Int J Epidemiol [Internet]. 1994;23(6):1145-1153. Available from: https://academic.oup.com/ije/article/23/6/1145/660338?login=true. doi: 10.1093/ije/23.6.1145

52. Letašiová S, Medve'ová A, Šovčíková A, et al. Bladder cancer, a review of the environmental risk factors. Environ Health [Internet]. 2012;11 Suppl 1(Suppl 1):S11-S11. Available from: <https://ehjournal.biomedcentral.com/articles/10.1186/1476-069X-11-S1-S11>. doi: 10.1186/1476-069X-11-S1-S11

53. Vermeulen SH, Hanum N, Grotenhuis AJ, et al. Recurrent urinary tract infection and risk of bladder cancer in the Nijmegen bladder cancer study. Br J Cancer [Internet]. 2015;112(3):594-600. Available from: https://pubmed.ncbi.nlm.nih.gov/25429525/. doi: 10.1038/bjc.2014.601

54. Anderson-Otunu O, Akhtar S. Chronic Infections of the Urinary Tract and Bladder Cancer Risk a Systematic Review. Asian Pac J Cancer Prev [Internet]. 2016; 17(8):3805-3807. Available from: <https://pubmed.ncbi.nlm.nih.gov/27644620/>.

55. Zaffanello M, Malerba G, Cataldi L, et al. Genetic Risk for Recurrent Urinary Tract Infections in Humans: A Systematic Review. J Biomed Biotechnol [Internet]. 2010;2010:321082. Available from: https://pubmed.ncbi.nlm.nih.gov/20379347/. doi: 10.1155/2010/321082

56. Lundstedt A-C, Leijonhufvud I, Ragnarsdottir B, et al. Inherited Susceptibility to Acute Pyelonephritis: A Family Study of Urinary Tract Infection. J Infect Dis [Internet]. 2007;195(8):1227-1234. Available from: https://pubmed.ncbi.nlm.nih.gov/17357062/. doi: 10.1086/512620

57. Turati F, Bosetti C, Polesel J, et al. Family history of cancer and the risk of bladder cancer: A case-control study from Italy. Cancer Epidemiol [Internet]. 2017;48:29-35. Available from: https://sci-hub.se/10.1016/j.canep.2017.03.003. doi: 10.1016/j.canep.2017.03.003

58. Murta-Nascimento C, Silverman DT, Kogevinas M, et al. Risk of bladder cancer associated with family history of cancer: do low-penetrance polymorphisms account for the increase in risk? Cancer Epidemiol Biomarkers Prev [Internet]. 2007;16(8):1595-1600. Available from: https:// pubmed.ncbi.nlm.nih.gov/17684133/. doi: 10.1158/1055-9965.Epi-06-0743

59. Gregorczyk-Maga I, Maga M, Wachsmann A, et al. Air pollution may affect the assessment of smoking habits by exhaled carbon monoxide measurements. Environ Res [Internet]. 2019;172:258-265. Available from: <https://www.sciencedirect.com/science/article/abs/pii/S0013935119300349?via%3Dihub>. doi: 10.1016/j.envres.2019.01.063

60. Hagenbjörk-Gustafsson A, Tornevi A, Andersson EM, et al. Determinants of personal exposure to some carcinogenic substances and nitrogen dioxide among the general population in five Swedish cities. J Expo Sci Environ Epidemiol [Internet]. 2013;24(4):437-43. Available from: https://www.nature.com/articles/jes201357. doi: 10.1038/jes.2013.57

61. Mobley D, Baum N. Smoking: Its Impact on Urologic Health. Rev Urol [Internet]. 2015;17(4):220-225. Available from: <https://www.ncbi.nlm.nih.gov/pmc/articles/PMC4735668/>.

62. Freedman ND, Silverman DT, Hollenbeck AR, et al. Association between smoking and risk of bladder cancer among men and women. JAMA [Internet]. 2011;306(7):737-745. Available from: https://pubmed.ncbi.nlm.nih.gov/21846855/. doi:10.1001/jama.2011.1142

63. Ma W, Zhang W, Shen L, et al. Can Smoking Cause Differences in Urine Microbiome in Male Patients With Bladder Cancer? A Retrospective Study. Front Oncol [Internet]. 2021;11. Available from: https://frontiersin.org/articles/10.3389/fonc.2021.677605/full. doi: 10.3389/fonc.2021.677605

64. Cancer Australia. Bladder cancer statistics in Australia [Internet]. [Strawberry Hills (NSW)]: Commonwealth of Australia; c2021. [revised 2020 Oct 20; cited 2021 Mar 9]. Available from: <https://www.canceraustralia.gov.au/affected-cancer/cancer-types/bladder-cancer/bladder-cancer-statistics-australia>

65. International Agency for Research on Cancer: Outdoor air pollution a leading environmental cause of cancer deaths [Internet]. Lyon: World Health Organization; 2013 [cited 2021 Mar 10]. 4 p. Report No.: PR 221. Available from: <https://www.iarc.who.int/wp-content/uploads/2018/07/pr221_E.pdf>.
